# From gangue to the fuel-cells application

**DOI:** 10.1038/s41598-020-76503-6

**Published:** 2020-11-18

**Authors:** M. Sherif El-Eskandarany, Sultan Majed Al-Salem, Naser Ali, Mohammad Banyan, Fahad Al-Ajmi, Ahmed Al-Duweesh

**Affiliations:** 1 Kuwait Institute for Scientific Research, 13109 Kuwait City, Kuwait; 2 Energy and Building Research Center, Kuwait City, Kuwait; 3 Environment and Life Sciences Research Centre, Kuwait City, Kuwait

**Keywords:** Environmental impact, Environmental sciences, Chemistry, Energy, Energy storage, Fuel cells, Renewable energy

## Abstract

Hydrogen, which is a new clean energy option for future energy systems possesses pioneering characteristics making it a desirable carbon-free energy carrier. Hydrogen storage plays a crucial role in initiating a hydrogen economy. Due to its low density, the storage of hydrogen in the gaseous and liquids states had several technical and economic challenges. Despite these traditional approaches, magnesium hydride (MgH_2_), which has high gravimetric and volumetric hydrogen density, offers an excellent potential option for utilizing hydrogen in automobiles and other electrical systems. In contrast to its attractive properties, MgH_2_ should be mechanically and chemically treated to reduce its high activation energy and enhance its modest hydrogen sorption/desorption kinetics. The present study aims to investigate the influence of doping mechanically-treated Mg metal with 5 wt% amorphous Zr_2_Cu abrasive nanopowders in improving its kinetics and cyclability behaviors. For the first time, solid-waste Mg, Zr, and Cu metals were utilized for preparing MgH_2_ and amorphous Zr_2_Cu alloy (catalytic agent), using hydrogen gas-reactive ball milling, and arc melting techniques, respectively. This new nanocomposite system revealed high-capacity hydrogen storage (6.6 wt%) with superior kinetics and extraordinary long cycle-life-time (1100 h) at 250 °C.

## Introduction

Low-carbon energy, solid-waste management, and climate change are considered the most challenging tasks facing humanity in the 21 st century. According to the fast-growing population and a rapid increase in the world economy, which are in conjunction with the intensifying growth in the heavy industrial sector, a dramatic increase in fossil fuel consumption is enoticeable^1^. As a natural consequence of using such hydrocarbon-based fuel, numerous harmful gaseous compounds (e.g., carbon dioxide, SO_2_, and NO_x_) are emitted^2^. Indeed, the unrestrained expansion of usage, such as pollutant-based hydrocarbon fuel, was responsible for producing a wide range of health and environment in worldwide^3^. Therefore, today's world is facing serious challenges for providing a sustainable source(s) of fuel suitable for generating clean energy^4,5^.

Hydrogen (H_2_), a well-known element, has been considered for an extended period as the best carbon-free energy carrier that can replace fossil fuels. It is very convenient, safe, multipurpose^6^, and when burned CO_2_ does not be produced. Moreover, H_2_, which has a high energy–density, can be obtained from renewable energy systems. The worldwide interest in hydrogen energy comes from its very high calorimetric value, with lower heating value (120 MJ kg^−1^)^8^, when compared to petrol (43 MJ kg^−1^)^8^. As a consequence, hydrogen is seen as the best carrier, in which it can be converted into the desired form of energy^7^. Based on these unique characteristics, and practicality found in H_2_, it is proposed as the future clean energy that could be started well before the oil reserves are depleted^4^.

Storage is considered as one of the most crucial roles for the hydrogen economy^9^. This area of research has been the subject of intensive work for many decades. Traditionally, hydrogen gas can be pressurized up to 700 bar and physically stored into cylindrical tanks made of high-strength polymer-based nanocomposites^4,10,11^. Undoubtedly, the lack of safely upon utilizing high-pressure hydrogen cylinders in the cart may limit the applications of such environmentally friendly fuel. Besides, a major drawback of the high-pressurized hydrogen gas tanks is its low storage densities^12^. Elsewhere, a mature alternative approach for hydrogen storage in the liquid state was proposed. One disadvantage of this approach is the massive volume of the liquid, which requires tanks with a size of almost three times more significant than currently used gasoline tanks^13^. More seriously than this, storage liquid hydrogen requires costly well-insulated cryogenic storage vessels to maintain the temperature below 20 K^12^. Concurrently, the gas-liquefication process of hydrogen itself is a very costly approach, in which consuming approximately 25–30% of the energy content of the stored hydrogen^14^.

In contrast to the two classic hydrogen storage approaches, Aceves and his team developed a new method for storing hydrogen, called cryogenic pressure vessel at the beginning of this century^12^. The idea behind this new storage approach was based on the combination of both high pressure and cryogenic methods, wherein a high-pressure (350 bar) cryogenic pressure vessel is operated at a low temperature (< 20 K). They claimed that hydrogen with this new storage method could be stored at a significantly higher density when compared with the other two methods^14^.

Aside from these unsafe and costly ways of hydrogen storage, a different approach to store hydrogen in its solid-state through the formation of metal hydrides such as magnesium hydride (MgH_2_) been receiving much attention for many years. Basically, the possibility of the formation of a metal hydride through the chemisorption concept dated back 1868, when T. Graham discovered that metallic palladium (Pd) readily charged by hydrogen^15^. As discovery followed the discovery, more than 50 metallic elements have shown beneficial properties for storing hydrogen by either physisorption or chemisorption processes^4^. Of these metals, Mg is considered the best candidate for hydrogen storage^16^. This is ascribed to its natural abundance, lightweight, and capability to store hydrogen up to 7.60 wt% (0.11 kg H_2_ L^−1^)^17,18^. Since the 1990s, reactive ball milling (RBM)^19,20^ has been used successfully for preparing MgH_2_ and MgH_2_-based nanocrystalline powders through high-energy ball milling Mg metal under H_2_ gas pressure^21,22–24^.

Unfortunately, MgH_2_ revealed significant drawbacks, such as sluggish kinetics and relative stable thermodynamic characteristics that restrict this attractive compound from real fuel-cell applications^16–18,22–25^. Within the last four three decades, considerable efforts have been paid in part to propose useful scenarios used to improving the kinetics behavior of MgH_2_ and to decrease its thermal stability (see, for example, Refs.^4,18,26^). Modification of the original crystal structure of MgH_2_ (tetragonal), which is very stable to metastable stable phases of orthorhombic^27^ and fcc^28^-MgH_2_ led to significant enhancement of the hydrogen storage behavior. Destabilization of β-MgH_2_ phase can be successfully performed by severe plastic deformation (SPD)^29^, using high-energy ball milling^30^, equal channel angular process (ECAP)^31^, high-pressure torsion (HPT)^32^, and cold rolling (CR)^33^ methods. The mechanical treatment of Mg/MgH_2_ leads to grain size refining and formation of metastable nanoscaled crystallites, as confirmed by Wagemans et al^34^.

Meanwhile and in parallel to the mechanically-induced approach, doping MgH_2_ with catalytic agents has been recognized as a practical scenario employed to improve the sluggish kinetics characteristics of this metal hydride. Since the 1990s, a long list of pure transition metals (TM) such as Ti, Zr, V, Nb, Fe, Co, Ni, and Mn^21,35–37^, and their alloys, exemplified by TiV^38^, CrTi^39^, TiMn_2_^40^, VTiCr^41^, and ZrNi_5_^42^. Almost all of these reported catalytic agents have led to significant merits, indexed by lower hydrogen sorption/desorption temperatures and faster hydrogenation/ dehydrogenation kinetics^4,18,26^. Moreover, nanocomposite systems such as MgH_2_/5Ni/5Nb_2_O_5_^43^, MgH_2_/5TiC^44^, and MgH_2_/10 big-cube Zr_2_Ni^45^ powders have shown superior kinetics behavior.

In contrast to that long list of long-range order catalysts with there beneficial effects, El-Eskandarany demonstrated the first study on employing short-range order material of an amorphous-Zr_70_Ni_20_Pd_10_ powder for improving the MgH_2_^23^. It is reported that employing such a metastable amorphous phase led to significant improvement in the kinetics characteristics of MgH_2_^23^. As yet, the literature reports on using amorphous/metallic glassy alloys are fewer^29,46,47^ when compared with the traditional alloys and compounds.

Apart from employing planetary-type high-energy ball mills, a unique design of the traditional Szigvari attritor ball mill^48^ has been recently utilized for the fabrication of nanocomposite MgH_2_/LiBH_4_ system^49–53^. This innovative method, termed as ball milling with aerosol spraying (BMAS), was proposed by Ding et al. in 2015^49^. In this BMAS process, ultrafine graphite (C) powders were prepared first through 6-h of high-energy ball mill under an argon atmosphere^49^. Commercial MgH_2_ powders were doped with 6 wt% of the pre-milled C powders, and then ball milled for 3 h, 6 h, and 12 h through the BMAS under continuous aerosol spraying of 2 M LiBH_4_/THF solution^49–53^. It was emphasized that the aerosol-liquid droplets solidified into nanoparticles when impinged onto the surfaces of MgH_2_ powders^49–51^.

They claimed that the formation of solid LiBH_4_ nanoparticles was attributed to the high local temperature (200–250 °C) that existed in the ball mill upon using high-milling speed^49^. At this expected temperature, the low-boiling point THF (66 °C) was vaporized instantaneously, leading to the formation of LiBH4 nanoparticles^52^. Further ball milling time led the LiBH_4_ nanoparticles to coat the surface of MgH_2_ powders first and be occluded into the powders with increasing the milling time^49,53^. Under these preparation conditions, in situ during ball milling to form MgB_2_ and LiH. The as-prepared nanocomposite LiBH_4_/MgH_2_ powders with 0.5 to 1 molar ratio possessed a good ability to reversibly release and absorb ~ 5.7 wt% H_2_ at 265 °C^49,51^.

Here we show, for the first time, the effect of cold-rolling followed with melt spinning and RBM on the hydrogen storage characteristics of solid waste (SW) Mg ribbons. For this study, an amorphous Zr_2_Cu alloy nanopowders, starting from SW metallic bulk materials, were prepared and employed as a novel metastable catalytic agent to enhance the hydrogen storage kinetics and cyclability of MgH_2_. The present study has been addressed to propose a solid-waste management concept via a multistep process, which is used for the first time to improve the performance and behavior of MgH_2_. Finally, the present work attempted to understand the doping mechanism of amorphous Zr_2_Cu upon catalyzation of MgH_2_.

## Results

### Cold rolled Mg-ribbons

**Figure 1:**
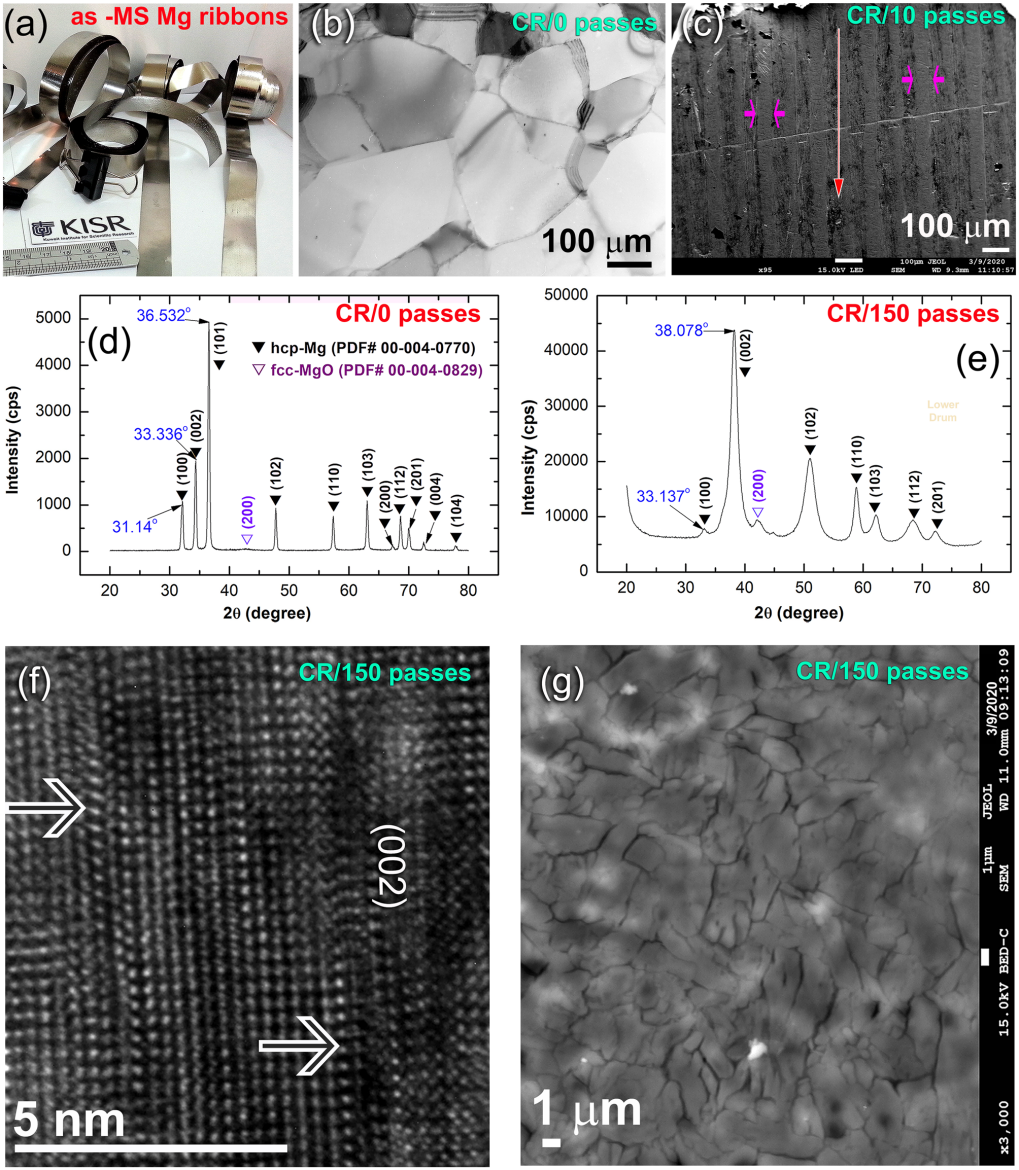
Structural characteristics of Mg ribbons after MS and CR processes. (**a**) Optical photograph of the starting MS–Mg ribbons, (**b**) BF/TEM image of the planner view for the initial Mg-ribbons, (**c**) low magnification SEM micrograph of the ribbons obtained after CR for 10 passes, (**d**,**e**) XRD patterns of MS–Mg ribbons obtained after CR for 0- and 10 passes respectively (**f**,**g**) FE-HRTEM image and SEM micrograph of the sample obtained after CR for 150 passes, respectively.

Figure 1a displays an optical photograph of Mg-ribbons obtained after the MS process. A TEM image in a low-magnification bright field (BF) mode for the planner view of an ion-milled polished sample for the MS Mg ribbon is presented in Fig. 1b. The ribbons were conglomerated of a mixture of large grains with an average grain size of ~ 425 nm (Fig. 1b). Initially, the Mg-ribbons revealed twin-free grain boundaries with no indication latter of lattice imperfections, as presented in Fig. 1b. This initial morphological feature was changed upon subjecting the ribbons for just 10 passes of CR, as shown in the low-magnification SEM micrograph in Fig. 1c. The as-CR ribbon sample, which obeyed the applied shear stresses during the CR process revealed micro-intimated bands resulted developed in a parallel direction of CR, as evidenced in Fig. 1c.

The XRD patterns of the starting MS Mg-ribbons (before CR), and after CR for 150 passes are presented in Fig. 1d,e, respectively. The as-MS sample exhibited sharp Bragg peaks corresponding to hcp-Mg (PDF# 00-004-0770) without evidence of any Bragg-peaks’ mismatches to the reported PDF data (Fig. 1d). This could be attributed to the absence of lattice imperfections in the MS–Mg ribbons. In contrast to the initially studied MS–Mg ribbons, two major hcp-Mg Bragg peaks, namely, (100) and (002), were shifted to the high-angle side, where the major crystallographic plane (101) had completely disappeared of the sample obtained after 150-passes of CR (Fig. 1e). This implies a severe lattice imperfection enforced during the CR process and the development of a very high level of the plane (002) of fiber texture^29^. The mechanically-induced lattice imperfection of this sample was confirmed by the FE-HRTEM technique. It turns out that the sample had revealed severe plastic deformation, as indicated by the formation of stacking faults presented in (002), as shown in Fig. 1f. The significant broadening, observed in the Bragg peaks of the as-CR sample for 150 passes (Fig. 1e) is attributed to the internal strain related to the existence of a high dislocation density and grain refinement. This led to outstanding imperfections in Mg-lattice, and formation of elongated fine grains (~ 2 to 14 μm in size), as implied in Fig. 1g.

### Reactive ball milling of as-CR Mg-ribbons

To investigate the synergetic effect of CR and RBM on the formation of nanocrystalline MgH_2_ powders, the as-CR Mg ribbons obtained after 150 passes (Fig. S1h) were snipped into small shots (Fig. S1i) and then RBM under 15 bar of H_2_, using a high energy tumbling mill. The effect of RBM time on the structural changes upon RBM of Mg-shots were monitored throughout XRD and HRTEM techniques. The XRD patterns of ball-milled shot-samples obtained after different stages of RBM are shown collectively in Fig. S2. The sample obtained after 3 h of RBM was mainly polycrystalline hcp-Mg metal, as characterized by the sharp Bragg-peaks related hcp-Mg metal (Fig. S2a).

During this early stage of milling, Mg-shots were subjected to the impact stresses generated by the ball milling media and started to defragment into small particles that had a fresh metallic surface of Mg. The mechanically-induced reactive milling took place between these fresh metallic surfaces of Mg and H_2_ to form a small volume fraction of β-MgH_2_ phase, as manifestly presented by new Bragg-lines corresponding to this metal hydride phase (Fig. S2a). Increasing the RBM time to 12.5 h led to further disintegration in the Mg-shots and hence larger volume fraction of the fresh-surfaces powders were obtained. These oxygen-free powders were capable to absorb greater H_2_ gas upon increasing the RBM process. Accordingly, the molecular fraction of MgH_2_ powders against the unprocessed Mg metal was dramatically increased. This is implied by the intensity increase related to the Bragg-peaks of β-MgH_2_ (Fig. S2b). We should emphasize that a small volume fraction of β-MgH_2_ (the most stable phase) was transformed into a metastable γ-MgH_2_ phase upon increasing the RBM time to 12.5 h. This is indicated by those new Bragg-peaks related to this metastable phase, which are indexed in Fig. S2b.

**Figure 2:**
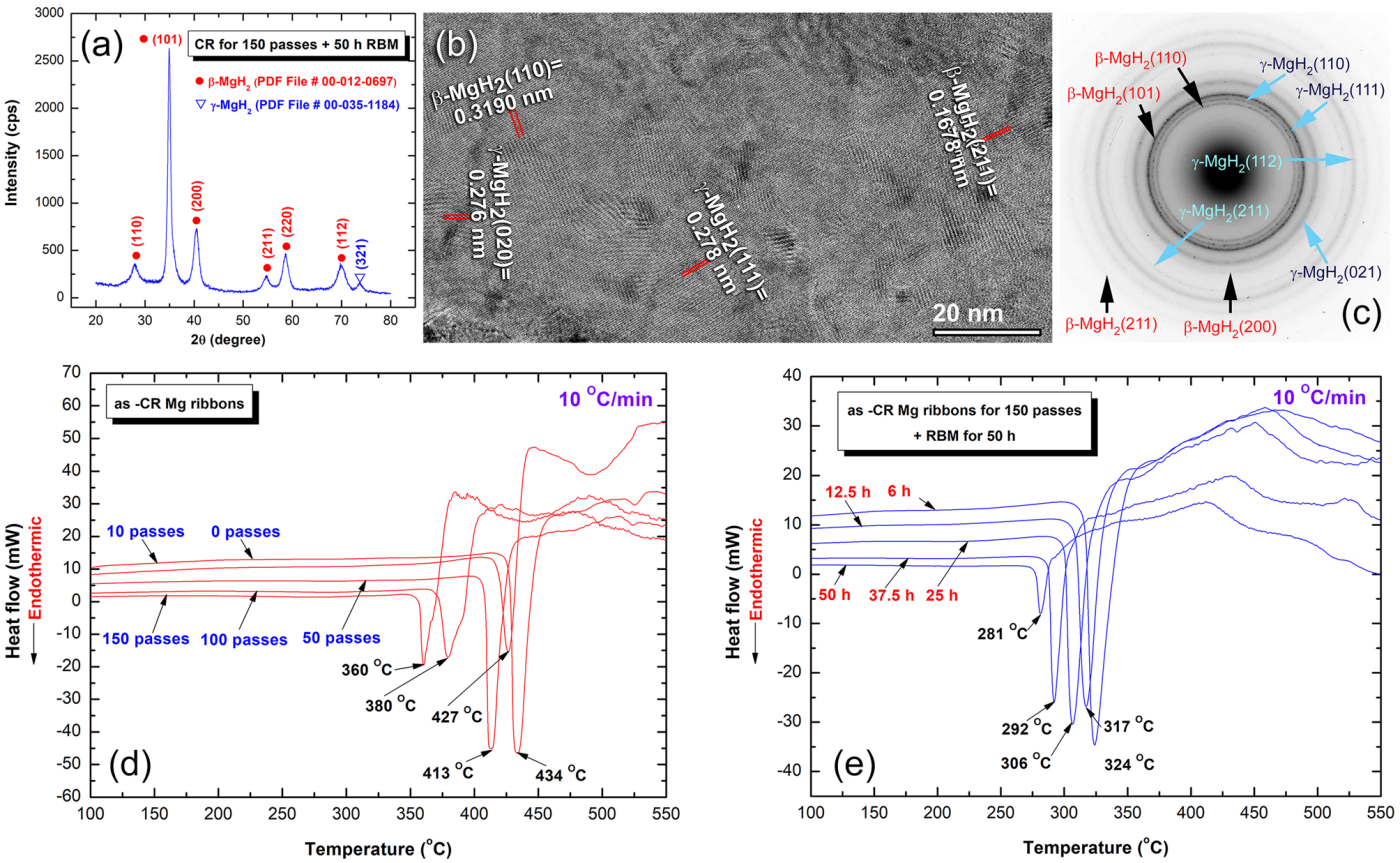
Structure and thermal stability of 150-passes CR Mg ribbons before and after RBM. (**a**) XRD pattern of 150-passes CR Mg ribbons, which were RBM for 50 h, (**b**) FE-HRTEM image, and (**c**) corresponding SADP of as-RBM samples for 50 h. The DSC thermograms of (**d**) as-CR ribbon samples obtained after 0, 10, 50, 100 and 150 passes and then hydrogenated at 300 °C under H_2_ gas pressure, and (**b**) as-CR sample for 150 passes after RBM for 6, 12.5, 25, 37.5 and 50 h.

The powders obtained after 25 h possessed a higher volume fraction of MgH_2_ powders with lower content of unprocessed hcp-Mg, as shown in Fig. S2c. Towards the end of RBM processing time (50 h), the Bragg-peaks related to Mg crystals were completely disappeared, and replaced by broad diffracted lines belonged to β-MgH_2_, as presented in Fig. 2a. This implying the completion of the RBM process for the formation of nanocrystalline MgH_2_ powders.

The FE-HRTEM image and corresponding selected area diffraction pattern (SADP) of the powders obtained after 50 h of RBM time are presented in Fig. 2b,c, respectively. The TEM analysis confirmed the formation of nano-dimensional grains (less than 20 nm) of β- and γ-MgH_2_ phases, as shown in Fig. 2b. The SADP revealed a continuous Dybe ring pattern related to β-, and γ-MgH_2_ phases, as highlighted in Fig. 2c.

The thermal characteristics of Mg ribbons obtained after different CR passes and then hydrogenated in a hydrogen reactor at 300 °C were investigated by the DSC technique in terms of decomposition temperature (T^de^). All the MgH_2_-ribbons without exception revealed a single endothermic event related to the decomposition of MgH_2_ phase, as shown in Fig. 2d. In general, these endothermic peaks were shifted to the low-temperature side upon increasing the CR passes (0–150 passes), as depicted in Fig. 2d. The starting MS–Mg ribbons (0 passes) displayed high value of T^de^ (434 °C), as shown in Fig. 2d. Increasing the CR to 10 and 25 passes, led to marginal improvement in T^dec^ values to 427 °C and 413 °C, respectively. When the ribbons were CR for 50 passes, an outstanding decrease of T^dec^ was attained. This is evidence by retreating the endothermic peak temperature (T_p_) to 380 °C (Fig. 2d). Towards the end of CR processing (150 passes), the H_2_ was released at a relatively low T^dec^ of 360 °C (Fig. 2d), signifying an outstanding effect of CR process to minimize the T^dec^ of MgH_2_.

Meanwhile, the DSC traces for as-CR Mg-ribbons for 150 passes and then subjected to RBM under 15 bar of H_2_ gas pressure for 6, 12.5, 25, 37.5, and 50 h are shown collectively in Fig. 2e. The thermal stability of this batch behaved differently when compared with the as-CR samples (without RBM). This was characterized by their rather low T^dec^ values that were detected at 324 °C, and 317 °C, for those RBM samples obtained after 6 h and 12.5 h, respectively (Fig. 2e). Further decreasing in T^dec^ was detected for the samples, which were RBM for 25 h (306 °C), and 37.5 h (292 °C), as depicted in Fig. 2e. Additional RBM time (50 h) was necessary to obtain more desired decrease in T^dec^ (281 °C), as manifested in Fig. 2e.

**Figure 3:**
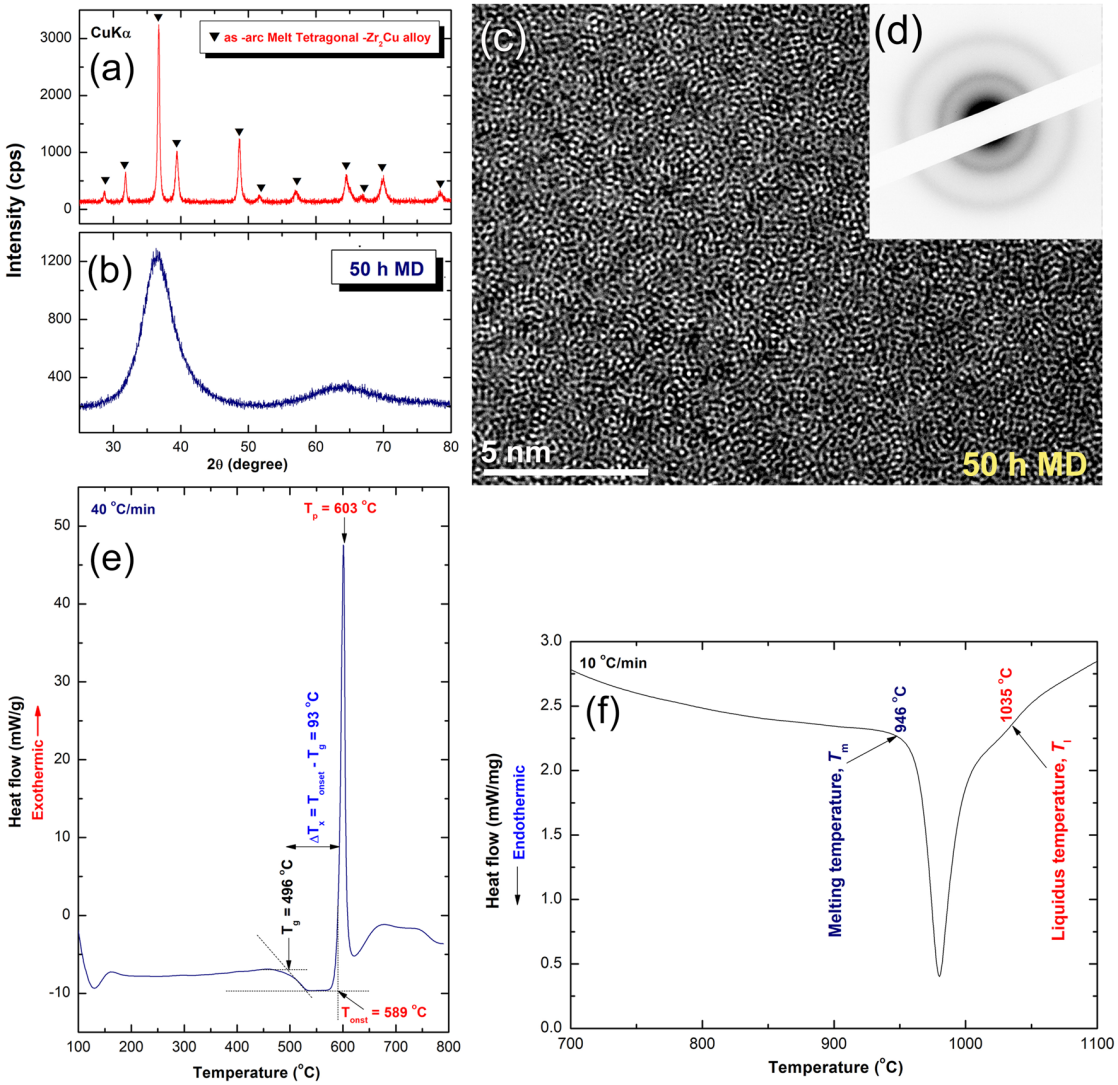
Structural and thermal stability of as-prepared a-Zr_2_Cu, used in the present study as a hydrogen storage modifier of MgH_2_. (**a**) XRD pattern of as-arc melt Zr_2_Cu alloy, and (**b**) as-arc melt alloy obtained after 50 h of MD. The FE-HRTEM image and corresponding SADP of the sample obtained after 50 h of MD time are shown in (**c**,**d**), respectively. The DSC trace and the DTA curve of the 50 h-RBM sample are displayed in (**e**,**f**), respectively.

Amorphous Zr_2_Cu nanopowders. Due to its excellent glass forming ability (GFA), good thermal stability and thermal conductivity, amorphous—(a) Zr_2_Cu binary system was selected to investigate its effect on the hydrogen storage and kinetic behaviors of as-prepared MgH_2_ powders. Amorphous-Zr_2_Cu phase was prepared through mechanical discording (MD) technique^54^, starting from intermetallic-Zr_2_Cu alloy, which was prepared by arc melting technique. The XRD pattern of as-disintegrated arc melt Zr_2_Cu alloy powders revealed a long-range order structure, as characterized by the sharp Bragg peaks presented in Fig. 3a. The analysis of these peaks confirmed the formation of high crystallinity Zr_2_Cu phase, being in a good agreement with the reported data^55^.

After 50 h of MD time, all the Bragg peaks corresponding to crystalline-Zr_2_Cu alloy disappeared and replaced by a broad diffuse halo of an amorphous phase (Fig. 3b). Formation of a-Zr_2_Cu phase was confirmed by FE-HRTEM, which manifesting a dense random close-packed structure with maze-like morphology (Fig. 3c). The related nano-beam diffraction pattern (NBDP) exhibited an amorphous-like halo-diffuse structure, as shown in Fig. 3d.

The thermal properties, indexed by glass transition temperature (T_g_), crystallization temperature (T_x_), supercooled liquid region (∆T_x_ = T_x_ − T_g_), and the enthalpy change of crystallizations (∆H_x_) of a-Zr_2_Cu phase, obtained after 50 h of MD time, were investigated through DSC technique. Where, its related melting behaviors, characterized by melting temperature (T_m_), liquids temperature (T_l_), and reduced glass transition temperature (T_rg_ = T_g_/T_l_) were investigated by differential thermal analysis (DTA) technique. Figure 3e displays the DSC traces of as-50 h MD a-Zr_2_Cu nanopowders. The thermogram possessed an endothermic event (T_g_) started at 496 °C (onset temperature) followed by an exothermic event (T_x_) shown at an onset temperature of 589 °C (Fig. 3e). At the first endothermic event (T_g_), which is a unique feature of metallic glassy alloys, the solid-amorphous phase tended to transform into a liquid phase. In the second event (T_x_), the liquid-amorphous phase (metallic glass) started to crystallize into a long-range order phase through a sharp exothermic peak, as presented in Fig. 3e. The onset (T_onset_) and peak temperatures (T_p_) of the crystallization peak were measured and found to be 589 °C and 603 °C, respectively (Fig. 3e). Where, the ∆H_x_, (the measured area under the exothermic peak) was − 6.88 kJ/mol. It should be emphasized that the broad ΔT_x_ value (93 °C), and its large ∆H_x_ (− 6.88 kJ/mol) implied that binary a-Zr_2_Cu system has a GFA. Besides, this amorphous system found in this metallic glassy system has good thermal stability, as authenticated by its high values of T_g_ and T_x_. The melting behavior of the system, characterized by DTA technique is shown in Fig. 3f. This a-phase revealed a single endothermic peak, started at 946 °C (T_m_), which was completed at 1035 °C (T_l_), as displayed in Fig. 3f. Here, the T_rg_ parameter was calculated found to be 0.48, indicated the good GFA of this system. The calculated value of this binary metallic glassy system, which was is used as a good indicator.

### Nanocomposite MgH_2_/5 wt% a-Zr_2_Cu powders

#### Structure and morphological properties

**Figure 4:**
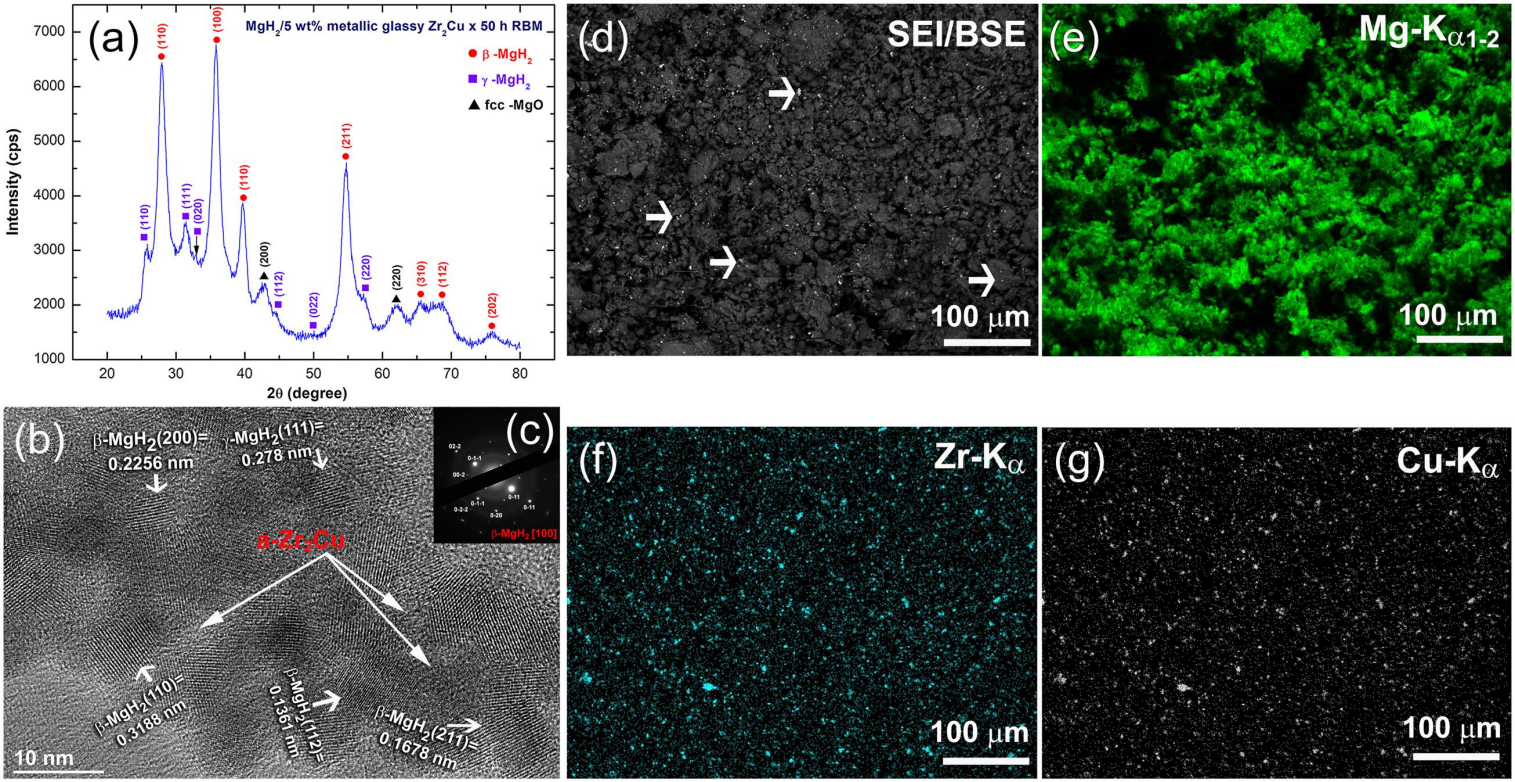
Structural and morphological properties of nanocomposite MgH_2_/5 wt% a-Zr_2_Cu powders, obtained after 50 h of RBM. (**a**) XRD pattern of the nanocomposite powders, (**b**) FE-HRTEM image, and (**c**) its related NBDP of the prepared nanocomposite powders. The low-magnification SEM micrograph of the powders is displayed in (**d**), where the corresponding EDS elemental mapping for Mg, Zr, and Cu are presented in (**e**,**f**,**g**), respectively.

The as-CR Mg-ribbons for 150 passes followed by high-energy RBM for 50 h under 15 bar of H_2_ were doped with 5 wt% a-Zr_2_Cu powders. The XRD pattern of as-RBM MgH_2_/5 wt% a-Zr_2_Cu powders, which were obtained after 50 h of the milling time is presented in Fig. 4a. The diffracted Bragg-lines shown in the figure were related to β- and γ-MgH_2_ phases, where the hump appeared in the baseline (2θ =  ~ 28°–45°) is matched well with a-Zr_2_Cu powders. Elsewhere, the low-intensity Bragg peak indexed by the black triangle symbols is corresponding to fcc-MgO (200) phase, which was formed during the preparation of the XRD sample outside He-glove box.

It is worth mentioning that the Bragg peaks of MgH_2_ presented in Fig. 4a revealed outstanding broadening, indicating the formation of nanocrystalline MgH_2_ powders with the amorphous powders. Comparing these Bragg-peaks with those presented in Fig. 2a, it can be declared the vital effect of a-Zr_2_Cu hard nanopowders for refining MgH_2_ powders upon further RBM time (50 h). The FE-HRTEM image of nanocomposite MgH_2_/5 wt% metallic glassy Zr_2_Cu nanopowders obtained after 50 h of RBM time is shown in Fig. 4b. The powders contained ultrafine nanocrystalline grains, which were ranging in size from ~ 6.5 to 8 nm, as presented in Fig. 4b. It can be concluded here that the hard metallic glassy Zr_2_Cu nanopowders, which played an important role as micro-milling media led to refining the metal hydride powders to the nano-level.

Analysis of the Moiré fringes revealed in Fig. 4b has indicated the presence of β- and γ-MgH_2_ phases. It is realized that MgH_2_ grains had random-crystallographic orientations, and hence, the direction of slip varies from one grain to another, as presented in Fig. 4b. Moreover, the featureless fine structure zones shown in the image was related to a-Zr_2_Cu, as displayed in Fig. 4b. Formation of MgH_2_/metallic glassy nanocomposite was confirmed by the NBDP that disclosed a diffuse halo ring of a-Zr_2_Cu overlapped with diffracted spots related to β-MgH_2_[100], as displayed in Fig. 4c.

To get more information about the effect of RBM on the distribution of a-Zr_2_Cu powders in the host MgH_2_ matrix, SEM/EDS elemental mapping was performed. The low-magnification SEM micrograph of MgH_2_ doped with 5 wt% of metallic glassy Zr_2_Cu powders and ball milled for 50 h of RBM time is shown in Fig. 4d, together with the corresponding EDS mapping of Mg (Fig. 4e), Zr (Fig. 4f), and Cu (Fig. 4g) elements. The fine powders obtained after this stage of RBM were agglomerated due to van der Waals forces to form larger aggregates, ranging in size between ~ 5 and 50 μm, as shown in Fig. 4d. These aggregated powders, which were MgH_2_ (Fig. 4e), adhered by ultrafine nano-lenses (~ 200 nm in size) of metallic glassy Zr_2_Cu, as displayed in Fig. 4f,g. It should be pointed out that the metallic glassy nanopowders were homogeneously distributed in the MgH_2_ powder matrix to give an average composition of MgH_2_/4.89 wt% Zr_2_Cu without severe fluctuation in composition beyond the submicron level.

#### Thermal properties

**Figure 5:**
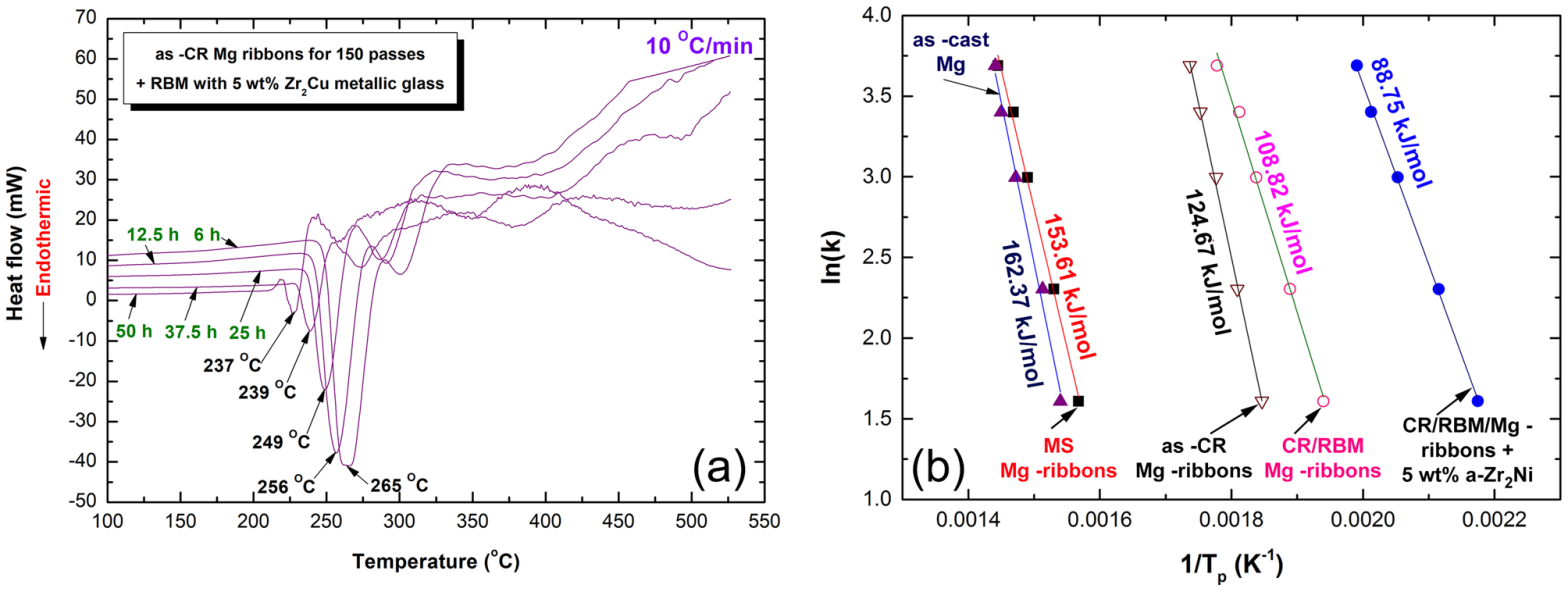
Thermal properties of nanocomposite MgH_2_/5 wt% a-Zr_2_Cu powders. (**a**) DSC traces of the powders obtained after RBM for 6, 12.5, 25, 37.5, and 50 h, (**b**) E_a_ curves of the as-cast Mg, as–MS ribbons for 50 passes, as RBM of the CR Mg-ribbons, and nanocomposite MgH_2_/5 wt% a-Zr_2_Cu powders obtained after 50 h of RBM.

However, T^dec^ values of the last two batches of CR ribbons and RBM of as-CR ribbon samples were within the range or even better than those previously reported values of MgH_2_ binary systems^12,18,26^, the present study has attempted to achieve further improvement of the thermal properties for this metal hydride system. To attain this purpose, we have employed our prepared a-Zr_2_Cu nanopowders as a novel nano-catalytic agent, used for reducing both of the T^dec^ and apparent activation energy (E_a_) of as-CR/RBM Mg-ribbons. After very short RBM times of 6 h and 12.5 h, T^dec^ were dropped to 265 °C and 256 °C, as shown in Fig. 5a. Further increase of RBM time to 25 and 37.5 h, led to an outstanding decrease in T^dec^ to the level of 249 °C and 239 °C, respectively (Fig. 5a). After 50 h of RBM, however, T^dec^ has not shown any significant changes, where it almost saturated at 237 °C (Fig. 5a) even after RBM for a longer RBM time (75 h).

The improved dehydrogenation kinetics of all samples in a He gas atmosphere was investigated by calculating the E_a_ of the decomposition endothermic reaction peaks, according to Arrhenius Equation:

1 $$ {\text{E}}_{{\text{a}}} = - {\text{RT ln}}\left( {\text{k}} \right) $$

where k is the temperature-dependent reaction rate (5, 10, 20, 30, and 40 K/min), and T_p_ is the peak temperature of the decomposition peaks. The value E_a_ of the reaction was determined by measuring the decomposition the T_p_ corresponded to the different heating rates (k) and then plotting ln(k) versus 1/T_p_, as shown in Fig. 5b. The best fit for the results was calculated by the least-square method. It follows from Fig. 5b that all data points lie closely on the same straight line.

The reference sample of as-melt/cast Mg, which was hydrogenated at 300 °C, possessed a very high E_a_ of 162.37 kJ/mol, as displayed in Fig. 5b. An improvement of E_a_ has hardly seen upon MS the cast alloy that still revealed a high E_a_ value (153.61 kJ/mol). In contrast to these untreated samples, a significant decrease in E_a_ (124.67 kJ/mol) was attained upon CR the Mg-ribbons for 150 passes, as presented in Fig. 5b. Superior improvement of E_a_ (96.82 kJ/mol) was earned after RBM the CR-Mg ribbons for 50 h (Fig. 5b). This value was furtherly decreased to 88.75 kJ/mol upon RBM with 5 wt% a-Zr_2_Cu nanopowders, as demonstrated in Fig. 5b.

Hydrogenation/dehydrogenation kinetic and cyclability. To realize the possibility of utilizing a hydrogen storage material for fuel cell applications, the materials must possess an excellent cyclability for achieving a large number of complete H_2_ uptake/release without failure or degradation in the kinetics and storage capacity. To ensure a good activation of the system and to break down the oxide layers formed on the powder surfaces, the examined sample was severely subjected to a continuous 10 hydrogenation/dehydrogenation cycles, taking place at 350 °C under the pressure of 35 bar/400 mbar. All of the prepared samples were then subjected to severe treatment for 180 continuous hydrogenation/dehydrogenation cycles, conducted at 275 °C under H_2_ pressure of 10 bar and 400 mbar, respectively.

The results of the cycle-life-time test of as-CR Mg ribbons for 150 passes are displayed in Fig. S3a. The measurements were conducted at 275 °C, under hydrogenation and dehydrogenation H_2_ pressure of 10 bar and 400 mbar, respectively. The hydrogen storage capacity of this sample maintained a saturation uptake and released values of 4.52 wt% H_2_ for almost 60 h (Fig. S3a). An obvious degradation in this value was observed after processing for a longer time (60–180 h), where the hydrogen storage capacity was monotonically decreased from 4.32 wt% to 3.83 wt% (Fig. S3a). Under these measurement conditions, the sample achieved 96 cycles/180 h (~ 1.89 cycle/h). On the other hand, the cycle-life-time kinetic behavior of as-CR Mg ribbon samples, obtained after RBM for 50 h under 15 bar H_2_ was improved, as shown in Fig. S3b. This is implied by a moderate increase in the number of cycles achieved during the test (113 cycles/180 h). However, the kinetics behavior of this sample did not reflect remarkable changes (~ 1.6 cycle/h), when compared with the as-CR sample (Fig. S3a).

The hydrogenation and consequent dehydrogenation kinetics measured at 250 °C for nanocomposite MgH_2_/x wt% a-Zr_2_Cu (x; 3, 7, and 10 wt%) powders, obtained after 50 h of RBM are shown in Fig. S4a,b, respectively. In general, all the samples revealed good absorption kinetics that were improved with increasing the volume fraction of a-Zr_2_Cu powders, as displayed in Fig. S4a. In contrast to the kinetics similarity between their hydrogenation kinetics, mixing the MgH2 powders with a large molar fraction of the catalytic agent led to a relatively decreasing in their hydrogen storage capacities, to be 6.2, 6.1, 5.9 wt% H_2_ upon using a-concentration of 3, 7, and 10 wt%, respectively (Fig. S4a). The corresponding dehydrogenation kinetics measured at 250 °C of this 3-sample are presented in Fig. S4b. The results showed a monotonical increasing the molar fraction of the amorphous phase led to enhance gas releasing kinetics, as shown in Fig. S4b. The MgH_2_/10 wt% a-Zr_2_Cu powders were able to release − 6.63 wt% H_2_ within 20 min, where it took 25 min to release − 6.46, and 6.22 wt% H_2_ when the amorphous concentration was 7 wt% and 10 wt%, respectively (Fig. S4b).

**Figure 6:**
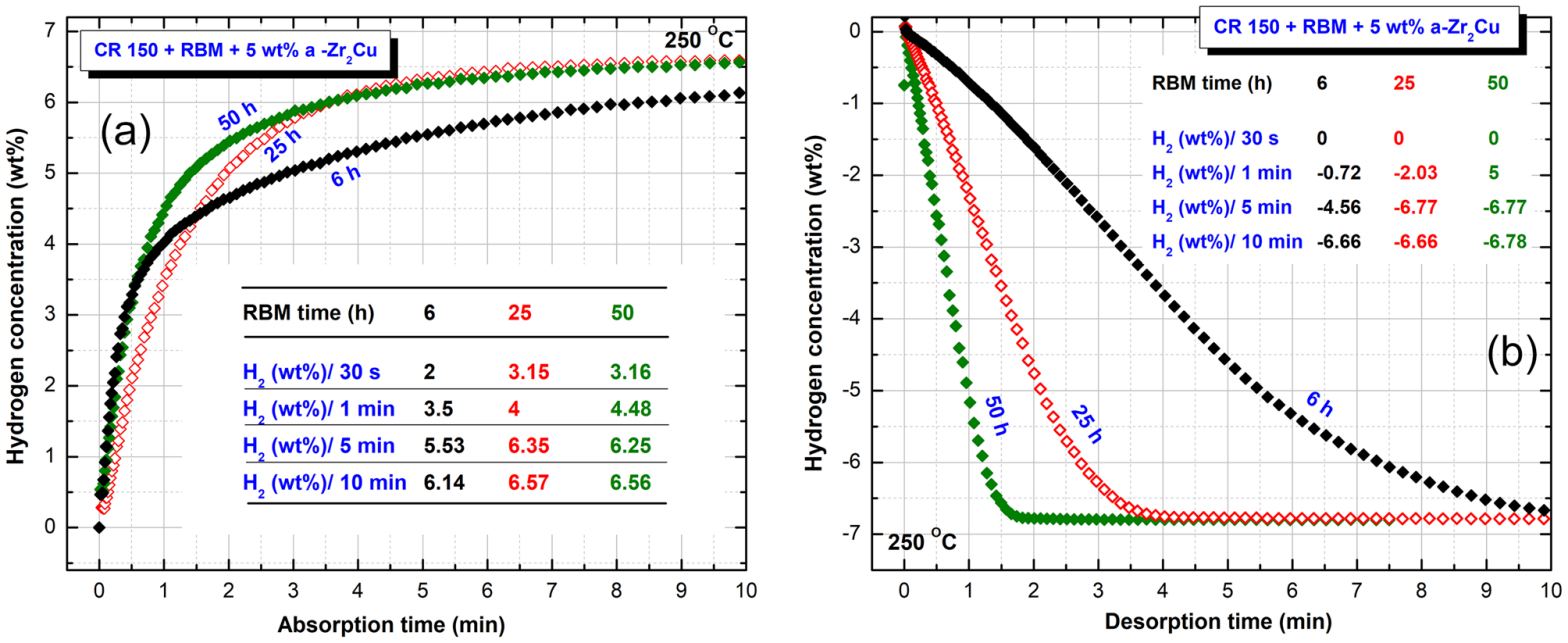
Effect of RBM time on the (**a**) hydrogenation, and (**b**) dehydrogenation kinetics behavior of MgH_2_/5 wt% a-Zr_2_Cu powders obtained after 6, 25, and 50 h. The measurements were conducted at 250 °C under uptake and release H_2_ pressure of 10 bar and 400 mbar, respectively.

The hydrogenation and consequent dehydrogenation kinetics measured at 250 °C for nanocomposite and MgH_2_/5 wt% a-Zr_2_Cu powders, which were obtained after selected RBM time (6, 25, and 50 h) are presented collectively in Fig. 6a,b, respectively. The hydrogenation kinetic behavior was investigated under 10 bar of H_2_, where dehydrogenation kinetics was measured under 400 mbar. The sample obtained after the early stage of RBM (6 h) was able to uptake 2 wt% of H_2_ after only 30 s of absorption time, as presented in Fig. 6a. Over the same absorption time (30 s), the sample obtained after 25 h revealed a significant increase in the H_2_ concentration (3.15 wt%), as displayed in Fig. 6a.

The hydrogen concentration value has not shown any remarkable change (3.16 wt%) for the 50 h RBM sample (Fig. 6a). Increasing the applied absorption time (1 min) led to enhance the hydrogenation kinetics of all samples, as depicted in Fig. 6a. The hydrogen uptake was 3.5 and 4 wt% of the sample obtained after 6 h and 25 h of RBM time. On the other side, the powder obtained after 50 h of RBM revealed a higher storage capacity of 4.48 wt%, as shown in Fig. 6a. Excellent kinetics behavior was realized upon increasing the absorption time to 5 min when all of the three samples reached higher H_2_ concentration values, as elucidated in Fig. 6a. Eventually, after 10 min of absorption time, the 6 h-, 25 h-, and 50 h- RBM samples earned to absorb high H_2_ concentration of 6.14, 6.57, and 6.56 wt% H_2_, respectively (Fig. 6a).

**Figure 7:**
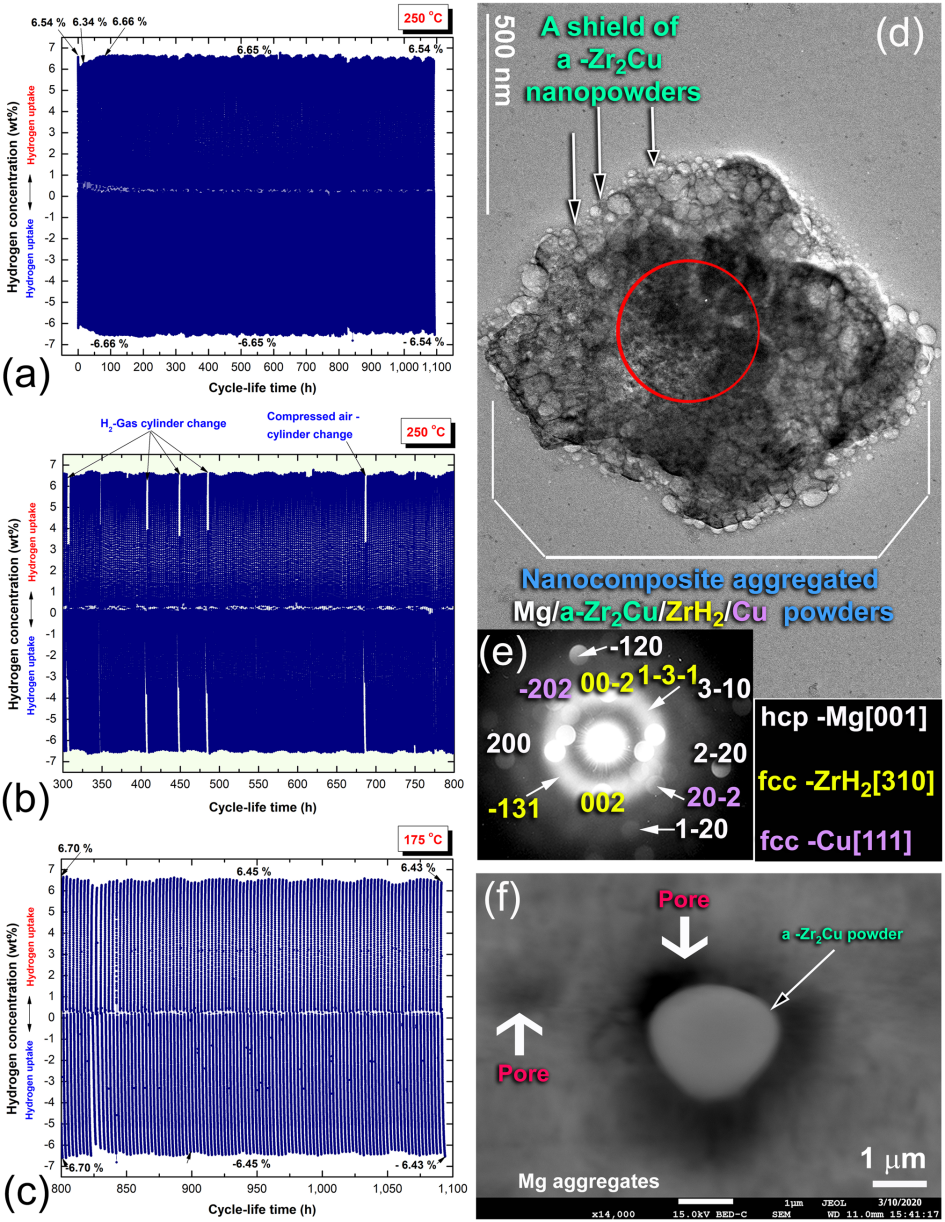
Cycle-life-time test of nanocomposite MgH_2_/5 wt% a-Zr_2_Cu powders obtained after RBM for 50 h. The complete cycle-life test conducted at 250 °C is presented in (**a**), where the middle portion of the curve (300–800 h) is presented in (**b**). The last 300 h-cycles, which were conducted at 175 °C is presented in (**c**). All the measurements were obtained over cyclic H_2_ pressure of 10 bar (hydrogenation) and 400 mbar (dehydrogenation). The BF/TEM image of the dehydrogenated powders obtained after ~ 1100 h is presented in (**d**) together with the (**e**) indexed NBDP related to –Zr_2_Cu, FE-HRTEM integrated with EDS elemental analysis through a beam diameter of ~ 5 nm the indexed zone shown in (**d**). The FE-SEM micrograph of the powders subjected to 1100 cycles is displayed in (**f**).

The cycle-life-time curve of the 50 h-sample is presented in Fig. 7a. This test was carried out for ~ 1100 h at 250 °C under a cyclic H_2_ pressure of 10 bar, and 400 mbar. Initially, the powders were activated at 350 °C through hydrogenation/dehydrogenation cycles undertook for 50 h under 35 bar/400 mbar of H_2_, respectively. After this treatment stage, the powders' hydrogen storage capacity increased from 6.54 to 6.66 wt%, as shown in Fig. 7a. In general, the powders were able to perform 1100 h continuously without noticeable kinetic degradation (Fig. 7a). Moreover, they maintained a high hydrogen storage capacity of 6.5 wt% H_2_ without recognizable deterioration in the H_2_ content, even after 1100 h, as shown in Fig. 7a.

The H_2_ uptake/release kinetics for the middle portion of the test (300–800 h) showed constant values (Fig. 7b) that are very closed to those shown in Fig. 6. Moreover, the powders had a constant reversible hydrogen storage capacity of ~ 6.6 wt%, as displayed in Fig. 7b. To understand the local crystal structure of the powders after the last hydrogenation cycle (800 h), the experiment was halted, where a few mg (~ 100 mg) of the hydrogenated powders were discharged from the sample holder in a He-glove box for TEM investigations.

**Figure 8:**
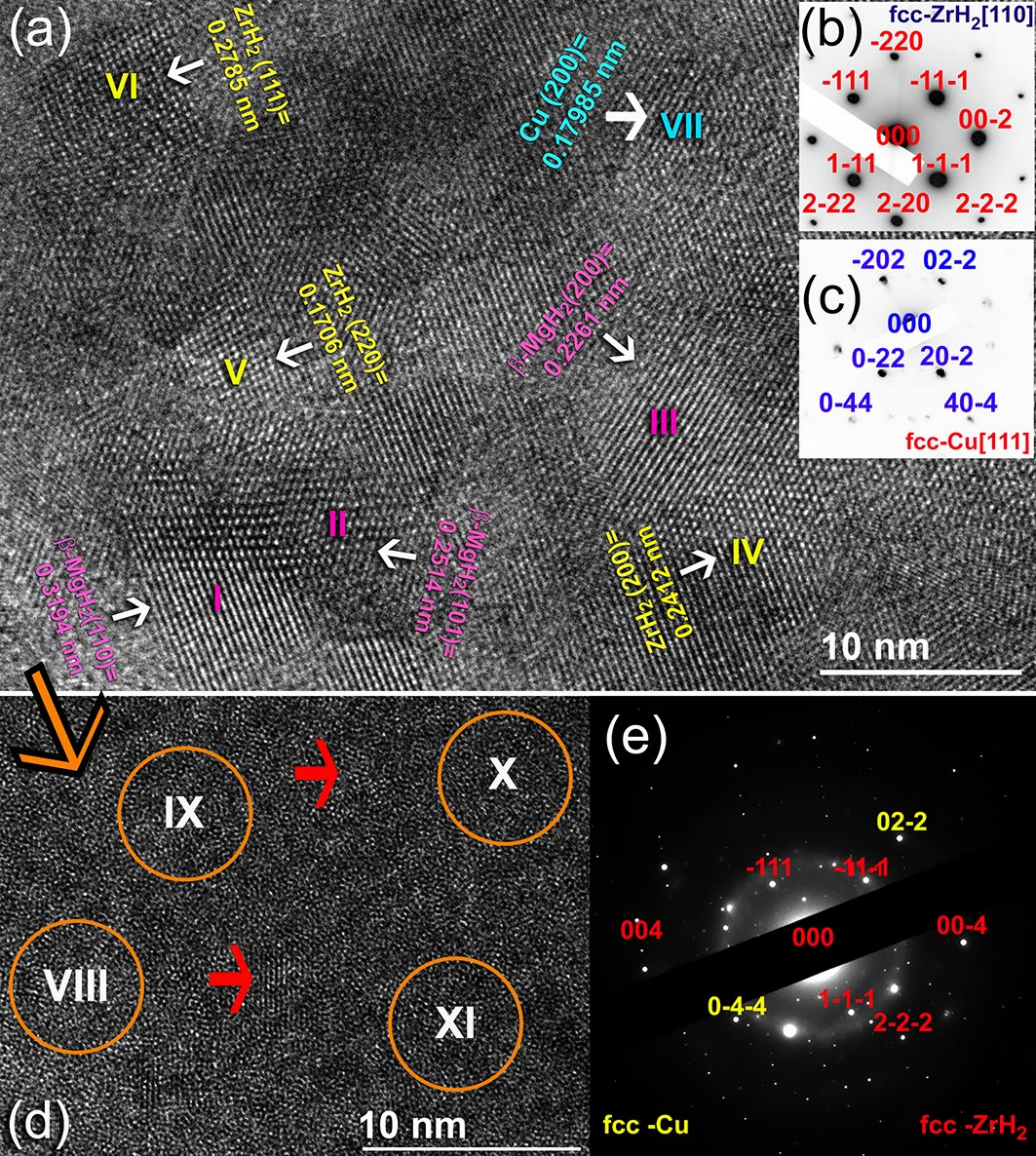
Local crystal characteristics of the 50 h-sample, which was obtained after the cyclic-life-time of 800 h at 250 °C under 10 bar of H_2_. (**a**) HRTEM image, (**b**) NBDP of zone I, and (**c**) NBDP of zone VII. The atomic resolution TEM image of the amorphous-zone and the corresponding NBDP are shown in (**d**,**e**), respectively. The circular zones indexed in (**d**) refer to the selected EDS elemental analysis regions.

The HRTEM images and corresponding NBDPs of this hydrogenated sample are collectively presented in Fig. 8. The powders composed of numerous nanocrystalline grains that revealed Moiré fringe images with diverse interplanar spacing (d). After this hydrogenation-cyclic time (800 h), the obtained β-MgH_2_ powders revealed fine sizes, ranging from ~ 6 nm to 10 nm, as indexed zones I, and III (Fig. 8a). Surprisingly, two extraneous phases rather than the tetragonal-MgH_2_ were detected in zones (IV, V, VI), and (VII), as indexed in Fig. 8a. It was first assumed that these two phases might be related to un-hydrogenated hcp-Mg powder and/or corresponding to a metastable Zr_2_Cu (fcc-Zr_2_Cu^56^) formed during this long-term of the cycle-life-time test. Careful analysis and measuring of the d spacing for 5 individual samples have led us to claim that a small volume fraction of a-Zr_2_Cu particles preferred to react with hydrogen to form δ phase of fcc-ZrH_2_^57^ (zones IV, V, and VI) and fcc-Cu^58^ (zone VII), as shown in Fig. 8a. This is implied by the very closed measured d-spacing values of both fcc-ZrH_2_ and fcc-Cu with the reference data. Besides, the spot analysis of NBDP taken from zone VI zone axis [110], indicated the formation of fcc-ZrH_2_ (Fig. 8b). Meanwhile, the sharp spots presented in Fig. 8c, taken from zone VII, is related to fcc-Cu [111].

To get more information about this partial phase separation, known as devitrification^59^ of binary a was used to characterize the featureless regions that existed between the crystalline grains (Fig. 8d). The examined area possessed featureless morphology, indicating that the amorphous-phase maintained its short-range, as implied by the halo-diffuse pattern presented in Fig. 8e. However, numerous fine-lenses (< 3 nm) of long-range order structure related to fcc-ZrH_2_, and fcc-Cu particles that were embedded into the amorphous matrix, as denoted by the red-arrows shown in Fig. 8d. This is evidenced by the spots that are overlapped with the amorphous ring presented in Fig. 8e. Local EDS analysis, using a beam diameter of 5 nm were conducted for 4 different individual samples to realize the chemical composition of a-Zr_2_Cu obtained after this partial phase separation. The atomic composition related to the examined zones (VIII–XI) indicated in Fig. 8d was varied from Zr_61_Cu_39_ to Zr_65_Cu_35_.

To examine the powder's ability on running cycle-life-time test at a lower temperature, the test was resumed after 800 h at 175 °C for 300 h, as displayed in Fig. 7c. The powders were able to get a good storage capacity of ~ 6.45 wt% H_2_, however, a serious degradation in the uptake/release desorption kinetics were observed, as shown in Fig. 7c. The BF/TEM image of the powders obtained after completion of a cycle-life-time of ~ 1100 h is presented together with the related NBDP in Fig. 7d,e, respectively. The fine powder particles were agglomerated to assemble equiaxed nanocomposite powders of Mg/a-Zr_2_Cu/ZrH_2_/Cu, with an apparent size of ~ 1.2 μm, as shown in Fig. 7d. It can be seen that a part of a-Zr_2_Cu particles have formed a fine shield surrounding the Mg-nanocomposite powders (Fig. 7e). The hard amorphous particles of this shield are believed to play a vital role as grain-growth inhibitors, restricting the metal hydride powders from growing during the cyclic process^57^. The NBDP corresponding to the indexed core zone Fig. 7d indicated that the powders composed of hcp-Mg, fcc-ZrH_2_, fcc-Cu, and a-Zr_2_Cu phases, as implied by the indexed spots and the halo-diffuse rings shown in Fig. 7e. Besides, the hard amorphous particles with their near spherical-like morphology tended to penetrate the rather soft surfaces of Mg powders upon applying hydrogen pressure (10 bar) at moderate temperature (250 °C) to create numerous “nano-hydrogen gates” that facilitated the simultaneous hydrogen charging/discharging processes, as presented in Fig. 7f.

**Figure 9:**
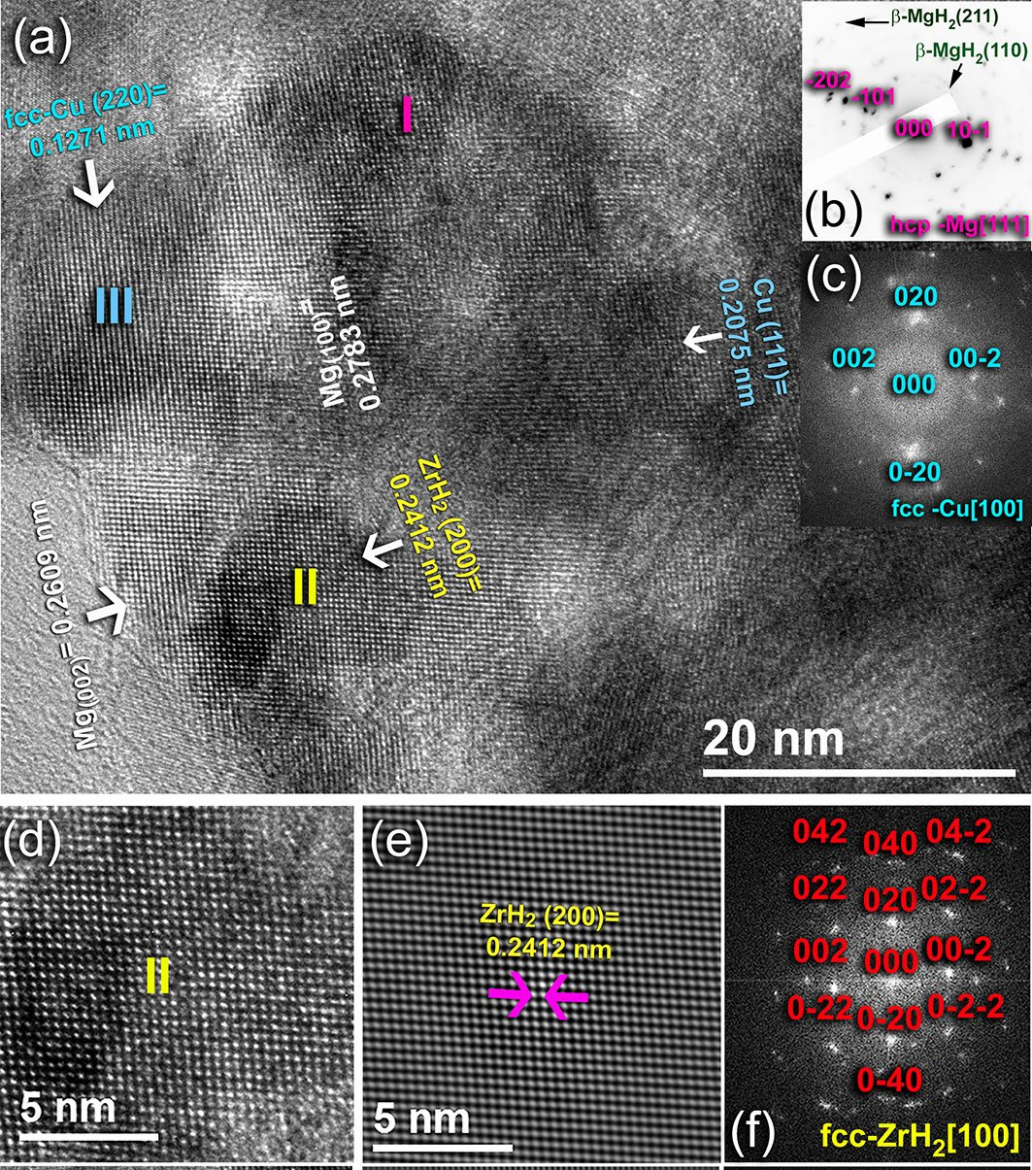
Local crystal characteristics of the 50 h-sample obtained after completion of the cycle-life-time test (1100 h). (**a**) HRTEM image, (**b**) NBDP of zone I, (**c**) FFT image of zone III, (**d**) atomic resolution TEM, (**e**) filtered TEM, and (**f**) FFT images of zone II.

Now, the question raised is, would fcc-ZrH_2_ simply decomposed into Zr metal and H_2_ gas upon subjecting to a cyclic dehydrogenation at a moderate temperature of 250 °C, or it may maintain its stability during the dehydrogenation test? Another question is, would the amorphous-Zr_2_Cu phase completely decomposed into ZrH_2_ and Cu-metals? To answer these questions, the last dehydrogenated sample obtained after ~ 1100 h (Fig. 7c) was obeyed to careful TEM analysis (Fig. 9). The FE-HRTEM image of as-desorbed sample revealed fringe images of many crystallites oriented in different orientations (Fig. 9a). The featureless fine regions presented in the image are related to the amorphous-Zr_2_Cu powder particles. Despite those small volume fractions for the undecomposed β-MgH_2_ particles, most of the powders contained nanocrystalline hcp-Mg metal with sizes of less than 10 nm (Fig. 9a). This is suggested by the overlapped rings and spots related to β-MgH_2_ and Mg, as shown in the NBDP (Fig. 9b). Existence of the fcc-ZrH_2_ phase was evidenced with the analysis of Moiré fringe-image shown in zone II (Fig. 9a). The atomic resolution TEM image and its corresponding filtered images of zone II oriented to [100] direction are presented in Fig. 9d,e, respectively.

The ZrH_2_ particle had a nanosized dimension of ~ 11 nm, with d spacing (0.2412 nm) matching well with (200). The existence of the ZrH_2_ phase was also evidenced by mean of spot analysis of the fast Fourier transform (FFT) presented in Fig. 9f. The d spacing value related to the fringe-image zone III in Fig. 9a (0.1271 nm) is referred to as the fcc-Cu metal particle, which is matching well with (220). This is also was confirmed by spot analysis of the FFT image (Fig. 9c) for fcc-Cu [100].The partial decomposition of a-Zr_2_Cu into ZrH_2_ and Cu phases was recently reported by Fadonougbo et al. when they pressurized the amorphous phase under 100 bar of hydrogen pressure^60^. The formed fcc-ZrH_2_ is decomposed throughout three accompanied endothermic reactions centered at 500, 730, and 800 °C^61^. Concerning this recent report, it may be inferred that the decomposition of ZrH_2_ phase is not expected to be accomplished during the dehydrogenation experiments of the present study, which was taken place at 250 °C.

**Figure 10:**
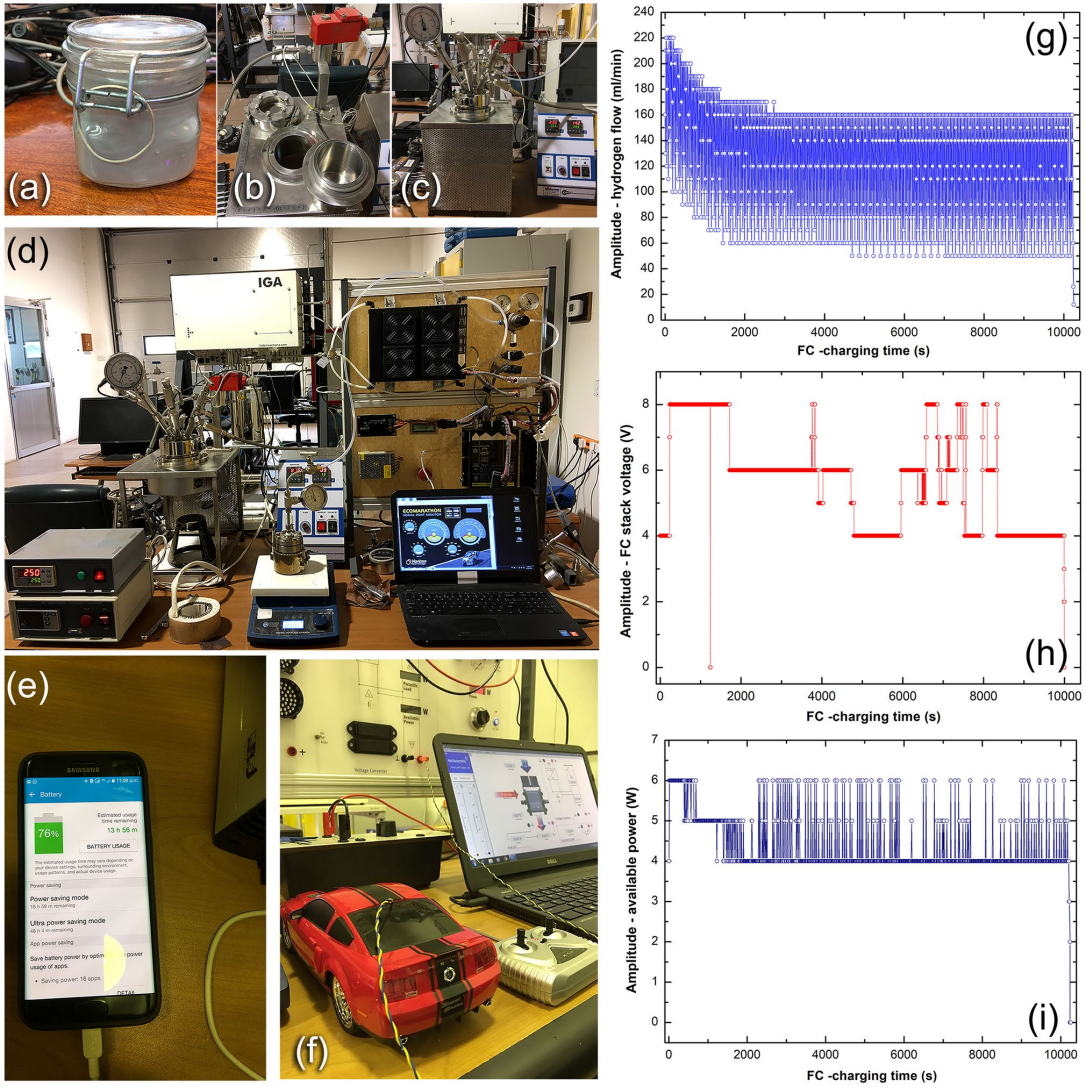
Experimental set up utilized for charging the battery of a cell phone device and a prototype of a battery car. (**a**) Nanocomposite MgH_2_/5 wt% metallic glass Zr_2_Cu powders obtained after 50 h, (**b**) tool-steel hydrogen reactor, (**c**) the reactor after charging with the powder and pressurized with 35 bar H_2_, (**d**) full-screen of the reactor and PEM-FC, (**e**) cell phone, and (**f**) prototype of a battery car. The dependence of H_2_ flow, voltage, and available power on the FC-charging time are displayed in (**g**,**h**,**i**), respectively. All of the photos shown in this figure have been obtained from The Nanotechnology Laboratory, Energy and Building Research Center (EBRC), Kuwait Institute for Scinetific Research (KISR), Kuwait.

Fuel cell applications. To meet the objectives of the present study, nanocomposite MgH_2_/5 wt% a-Zr_2_Cu powders obtained after 50 h of RBM were used as solid-hydrogen fuel required to operate 500 W/15 A proton exchange membrane (PEM) fuel cell (FC). The generated electric power was utilized to charge the battery of a cell phone device and to operate a prototype battery car (3 V) through 5 V voltage regulator. To attain this target, 300 g of the powders (Fig. 10a) were charged into a customized hydrogen storage tool-steel tank, as displayed in Fig. 10b. A pressure gauge (50 bar) and a high-pressure ball gate valve systems were perfectly installed into the tank cap’s to monitor the hydrogen gas released and charged (Fig. 10c). The system was then tightly sealed and evacuated to the level of 10^–2^ bar before pressurizing it under 35 bar of H_2_. The hydrogen reactor tank was connected to a PEM-FC unit, controlled and operated through software, as depicted in Fig. 10d. The data related to the H_2_ flow rate, voltage, and available power obtained during this test were stored. Figure 10e,f display the experimental set up utilized for charging the battery of a cell phone device and a prototype of battery car, respectively. Figure 10g presents the relationship between the FC-charging time of a cell phone battery and the H_2_ flow released from the nanocomposite powders upon heating the powders at 250 °C. On the other hand, the relationship between the FC-charging time and (1) FC stack voltage, and (2) available power are shown in Fig. 10h,i, respectively. The MgH_2_ phase in the nanocomposite system was decomposed within the first 500 s of the battery charging time. This was evidenced by monitoring the pressure increase of the released hydrogen that was saturated at a value of 25 bar, as indicated in Fig. 10c. To ensure the absence of unexpected backward reaction between the Mg powders and the liberated hydrogen, the heating system was completely discontinued. Consequently, the hydrogen released was then evacuated with a conventional rotary pump and delivered continuously through pipelines to the PEM-fuel cell. The hydrogen gas evacuated to the PEM-FC system was evidenced by a remarkable continuous slump in the H2 pressure value inside the reactor. Facilitating a rotary pump between the hydrogen reactor and PEM-FC system had beneficial assisted in delivering the gas consistently (~ 130 ml/min) to the PEM-FC, as presented in Fig. 10g. As a consequence, such a constant flow rate of H2 led to producing electricity with constant voltage (4–8 V), and power (5 W), as demonstrated in Fig. 10h,i, respectively. Towards the end of the experiment (10,000 s), the H_2_ pressure measured inside the tank reached to 1 bar. This indicated the completion of hydrogen releasing and end of the battery charging process. Details of these experiments can be found in Ref.^40^.

## Discussion

Based on the finding in our study, solid-waste Mg, Zr, and Cu metals were utilized for tailoring high-hydrogen storage capacity (above 6.5 wt% H_2_) of MgH_2_/5 wt% a-Zr_2_Cu nanocomposite powders, using RBM technique. The new procedure was applied to maximize the performance capability of Mg to uptake/release H_2_ fast. This proposed method can be classified into four subsequent stages described thus.

### Stage I

This stage starts by introducing severe plastic deformation of as-MS-Mg ribbons (Figs. 1a,b) through CR approach (Fig. S1). The CR technique, which is based on the principle of the formation of a highly fragmented and disoriented structure (Fig. 1f), obtained by very large deformations, has led to improve the hydrogenation/dehydrogenation kinetics of the disordered hcp-Mg nanocrystalline shots CR for 150 passes (Fig. S3a). On the basis of the present results, CR–Mg ribbons have faced severe degradation in their H_2_ storage capacity with apparent downgrading in the corresponding kinetics, as shown in Fig. S3a. This might occur due to the disability of Mg grains to maintain their nanodimension characteristics during the cycle-life-test conducted at 275 °C (Fig. S3a). Accordingly, further mechanical treatment of CR–Mg shots was necessary to magnify the effect of mechanical deformation with using high-energy RBM operated under 50 bar of H_2_.

### Stage II

During this second step of mechanical treatment, the Mg shots were severely subjected to different types of lattice imperfections, such as point defects and vacancies that led to disintegrate the powder particles along their grain boundaries into finer nanocrystallites, as shown in Fig. 2b. Formation of these nano-dimension MgH_2_ crystallites led to an entire decrease in the path for hydrogen diffusion. This can be realized by the enhancement achieved in the kinetics and cyclability of the powders obtained after 50 h of RBM (Fig. S3b). Moreover, the RBM powders revealed lower E_a_ (~ 109 kJ/mole) when compared with CR–Mg ribbons (~ 125 kJ/mole), as shown in Fig. 5b. In fact, the hydrogen storage properties, indexed by the storage capacity and kinetics cyclability (Fig. 3b) of the end product for this second stage do not match the objectives of the present work. Therefore, further efforts were dedicated to decrease the powders size through introducing hard nanoscaled milling media of a-Zr_2_Cu.

### Stage III

In this stage, the as-RBM MgH_2_ powders obtained after 50 h were doped with abrasive nanopowders media of a-Zr_2_Cu, and then RBM for 50 h. The selection of this amorphous phase was based on its high thermal stability, which is indexed by its high T_x_ (589 °C), ΔT_x_ (93 °C), and ∆H_x_ (− 6.88 kJ/mol) values, as shown in Fig. 3e. Choosing of metastable materials with high T_x_ as catalytic agent ensures the absence of crystallization during the kinetics and cycle-life-time measurements that take place at 250 °C. In addition to this, Zr-based amorphous and metallic glassy materials possess very high Vickers hardness values that lead to effective fragmentation of the MgH_2_ powders (Fig. 4a). In addition, these hard metastable nanopowders can act as grain growth inhibitors during the kinetics measurement. Moreover, Zr-based amorphous alloys with their excellent thermal conductivity can enhance the poor thermal conductivity of MgH_2_ powders that leads to improve their hydrogen storage capacity and kinetics behavior, as shown in Fig. 6.

The a-Zr_2_Cu nanopowders, which were homogeneously distributed into MgH_2_ powders (Fig. 4b,d–g) had an excellent effect on decreasing of E_a_ to the level of ~ 89 kJ/mol, as shown in Fig. 5b. The superior hydrogenation/dehydrogenation kinetic behavior of MgH_2_/5 wt% a-Zr_2_Cu powders examined at 250 °C can be considered as one of the best known MgH_2_-systems.

It should be emphasized that the small volume fractions of nanosized fcc-ZrH_2_ and fcc-Cu phases (Figs. 8a and 9a) that were dissociated from Zr_2_Cu had additional beneficial effects on improving the kinetics and cyclability of the present system. Likewise TiH_2_, NbH, and VH powders ZrH_2_ lead to change the electronics state of MgH_2_ powders, leading to enhance cyclability and gas uptake/released kinetics. Beside a-Zr_2_Cu, the existence of nanosized Cu metal in MgH_2_ matrix had a superior effect on increasing the thermal conductivity of MgH_2_, resulting in an improvement of their storage kinetics characteristics (Fig. 6).

## Conclusions

In summary, solid-waste Mg metal was employed to prepare Mg-ribbons through melt spinning approach, where solid-waste Zr and Cu metals were treated to fabricate a-Zr_2_Cu alloy powders via high-energy ball milling. The Mg-ribbons were plastically deformed with the CR technique to prepare thin strips that showed modest hydrogenation/dehydrogenation kinetics behavior. These ribbons revealed better kinetics and cyclability upon high-energy ball milling for 50 h under 15 bar of H_2_. Superior hydrogen uptake/release kinetics of 6.6 wt%/5 min at 250 °C was attained over RBM under with 5 wt% a-Zr_2_Cu nanopowders for 50 h. This new nanocomposite system possessed excellent cyclability at 175–250 °C to achieve 1100 h of cycle-life-time under hydrogen uptake and release the pressure of 400 mbar and 10 bar, respectively. Partial decomposition of a-Zr_2_Cu into fcc-ZrH_2_ and fcc-Cu was observed during the cyclic hydrogenation process, they maintained their chemical composition upon dehydrogenation cycles. The present work pointed out to the importance of three combined factors that have a great effect of enhancing the hydrogenation/dehydrogenation kinetics, and cyclicality of MgH2, i.e. (1) cold-rolling, (2) high-energy ball milling, (3) mechanically-induced doping with a-Zr_2_Cu catalytic agent, and (4) existence of ZrH_2_ and Cu.

## Experimental

### Preparations of the feedstock materials

The materials used as feedstock in this work were batches of 10 kg of pure Mg chips (25–35 mm in length and ~ 20 mm in thickness, 99.5 wt%), acquired from Shanghai Xinglu Chemical Technology Co. Ltd. (Shanghai-China), 3 kg of pure Zr scrap rods (20 cm in length and 1 cm in diameter, 97.5 wt%) produced by India MART InterMESH Ltd. and 2 kg of Cu scrap-clad laminate (different size variety with a purity of 99.99 wt%) sourced from Jiangmen Longxing Electronic Materials Co., Ltd (China). All of the as-received scrap metals were firstly sonicated in a cold acetone bath for 15 min to ensure the removal of all machine oil coolants from their surfaces. After this, they were rinsed with pure ethanol that took place before drying in an oven at 180 °C for a continuous period of 18 h. This primer treating step was necessary to remove all of the undesired hydrocarbon contaminants from the materials’ surfaces.

Figure S1 summarizes the conversion of SW-Mg machining chips into macroscale Mg-shots. In this process, an amount of 300 g of oil-free Mg-chips and 100 g of Cu plates were then charged independently in a graphite crucible and placed in a conventional melting/casting machine. The melting section was firstly evacuated and depressurized to 10^–5^ bar before being filled with pure argon (Ar, 99.99 wt%) gas. Then, the melting process was performed at 750 °C and 1200 °C for Mg and Cu, respectively. Three continuous melting cycles were applied for each metal. In general, during the melting process, 100 ml of Ar gas was introduced frequently (5 to 8 times), to ensure the purification of the molten Mg and Cu. Towards the end of the melting process, the bottom part of the graphite crucible was opened where the molten metal was sunk into a cylindrical graphite crucible at a temperature of almost 400 °C (for Mg) and 900 °C (for Cu). The system was kept for 9 h before removing the metal ingot from the crucible. The elemental analysis of the cast Mg showed the material to be pure (99.88 wt%) with less than 0.11 wt% oxygen (O_2_) and 0.01 wt% carbon (C), as characterized by inert gas fusion, and thermal conductivity detection test techniques, respectively.

Elsewhere, the Cu ingot revealed high purity of 99.994 wt% with O_2_ and C contamination of 0.004 and 0.002 wt%, respectively. A certain amount (~ 250 g) of as snipped Zr pieces were placed in a water-cooled copper-hearth of an arc melter (Edmund Bühler, Germany). The refining process of Zr scrape was conducted using titanium (Ti) getter under 0.8 bar of pure Ar and 450 A. The chemical analysis of the obtained Zr button, possessed high purity of 98.7 wt%, with hafnium (Hf), O_2_ and C contamination of 0.8, 0.3, and 0.2 wt%, respectively.

About 5 g of small pieces (~ 30–40 mm^3^) of the as-cast Mg ingot were inserted in a quartz tube and fixed in PA 500 melt spinner (MS) machine, provided by Edmund Bühler, Germany. A single Cu-wheel drum, rotated at 5000 rpm, was used to provide a rapid quenching of the melt. The MS process was first evacuated to the level of 10^–6^ before introducing pure helium, He (99.99 wt%) gas to the quartz-crucible and MS chamber. Then, the crucible was pressurized with 3 bar of He gas, and the chamber was maintained under 0.8 bar of He. The induction melting was taken place at 750 °C. The molten Mg fluid was enforced to travel into the hole existed in the bottom of the quartz-crucible upon pressurizing the crucible with an extra 2 bar of He gas. The Mg-droplets passed from the hole were rapidly quenched with a very high cooling rate generated by the rotated Cu-drum. The end product of MS Mg was in the shape of ribbons of ~ 1.3 cm in width, ~ 300 cm in length, and ~ 0.5 cm in thickness. Details of these experiments.

The as MS ribbons were cut into smaller strips of almost 20 cm in length and then subjected to cold rolling (CR) for 150 passes, using a conventional two tool steel drum cold roller machine. The strips were warm pressed at 150 °C for 5 min after every 10 passes using a two plate warm press to minimize brittleness of the Mg strips that was occurred during the CR process. The CR-Mg-strips were elongated by approximately 112% after the completion of 150 passes. The average thickness of the as-CR Mg strips was reached to 108 μm after CR. The CR Mg strips were cut into short ribbons of approximately 4 to 10 mm in length, 0.5 cm in width. These snipped strips were cleaned with acetone and ethanol and then dried in an oven at 150 °C overnight. The as-snipped Mg shots were sealed under He gas atmosphere and kept in an argon glove box.

The arc melting technique was used to prepare a master alloy of Zr_67_ (74.17 wt%) Cu_33_ (25.83 wt%) were used in this study as a catalytic agent for MgH_2_. The recovered Zr and Cu pieces with a mass of 74.17 g and 25.83 g resulted in the nominal composition of Zr_2_Cu, which were placed in the water-cooled copper hearth of the arc melter. The melting process started under 0.8 bar of pure Ar, with 450 A. To ensure the homogeneity in the chemical composition, the molten button was turned over and remelted for 5 times. The chemical analysis of prepared Zr_2_Cu indicated the formation of pure master alloy composited of Zr (74.08 wt%), Cu (24.96 wt%), Hf (0.8 wt%), O_2_ (0.04 wt%), and C (0.03 wt%).

### Sample preparations

#### Nanocrystalline MgH_2_ powders

An amount of 50 g of Mg shots and 250 tool steel balls were charged into a cylindrical vial made of tool steel and sealed together in a glove box (UNILAB Pro Glove Box Workstation, mBRAUN, Germany) under pure (99.99 wt%) He gas atmosphere. The ball-to-powder weight ratio was 36:1. The sealed vial was then evacuated to the level of 10^–3^ bar. The vial was then pressurized with 15 bar of hydrogen gas (H_2_) and mounted on a tumbling roller mill. The RBM process was conducted at room temperature, using a milling speed of 250 rpm. To understand the mechanism of mechanically-induced gas–solid reaction taking place during the RBM process, the tumbling mill was interrupted after selected milling time (3, 6, 12.5, 18, 25, 37.5, and 50), where the milled powders were completely discharged from the vial in the He-glove box. The RBM process was resumed with fresh batches of CR Mg ribbons using the same milling parameters.

Amorphous Zr_2_Cu nanopowder particles. The as-prepared master alloy of Zr_2_Cu was disintegrated into small pieces (~ 2.5 cm) and then charged in a conventional lab-type disc mill. The disc-milled powders were classified through a sieving system to collect fine powders of ~ 50 μm in diameter. Whereafter, the disintegrated powders were introduced to a planetary mill vial (500 ml in volume) made of tool steel alloy vial and well-sealed under He atmosphere, with 75 tool steel balls (11 mm in diameter) inside the glove box. The ball-to-powder weight ratio was kept at a ratio of 20:1. The vial was installed on a planetary-type ball mill (PULVERISETTE 5, Fritsch, Germany), where the ball milling (BM) process was carried out at a rotation speed of 250 rpm for 25, and 50 h at ambient temperature. The milling process was halted after the desired BM time, where a small sample of the powders was taken from the vial for several analyses. Thereafter, the milling process was consequently resumed under the same operating conditions.

#### Nanocomposite Mg/5 wt% amorphous-Zr_2_Cu powders

The as-prepared MgH_2_ powders that were obtained after 50 h of the RBM time were firstly doped by 5 wt% of amorphous (a) Zr_2_Cu powder and manually mixed inside under He atmosphere inside the glove box. The mixture was charged into three tool steel vials (150 ml in volume) made of tool steel alloy. A number of 50-tool steel balls (11 mm in diameter) were used as milling media, using a ball-to-powder weight ratio of 40:1. In the present study, a gas-temperature-monitoring system, supplied by evico magnetic, Germany, was utilized to follow the progress of the RBM process that was carried out through a high-energy ball mill (Planetary Mono Mill PULVERISETTE 6, Fritsch, Germany). Details of these experiments were reported elsewhere^23^. The RBM process was discontinued after the desired RBM time to take samples used for different analyses.

### Sample characterizations

#### Crystal structure

The crystal structures of all samples were investigated by X-ray diffraction (XRD) with CuKα radiation, using 9 kW Intelligent X-ray diffraction system, provided by SmartLab-Rigaku, Japan^24^. The local structure of the synthesized materials was studied by 200 kV-field emission high-resolution transmission electron microscopy/scanning transmission electron microscopy (HRTEM/STEM) supplied by JEOL-2100F, Japan, and equipped with Energy-dispersive X-ray spectroscopy (EDS) supplied by Oxford Instruments, UK^23^. A Cryo Ion Slicer Machine (IB-09060CIS) supplied by JEOL-2100F, Japan was used to prepared TEM samples of as-CR Mg ribbons. Details of the XRD characterization was recently published elsewhere^28,46^.

#### Morphology and elemental analysis

The morphological characteristics of the milled powders were investigated through a field emission scanning electron microscope (FE-SEM) using a voltage of 15 kV in a JSM-7800F model JEOL Japan microscope^25^. The elemental analysis was investigated by energy-dispersive X-ray spectroscopy (EDS, Oxford Instruments-UK) system interfaced with the FE-SEM^24^. More details are presented in Refs.^25,40^.

#### Thermal stability

Differential scanning calorimeter (DSC), provided by Setaram–France, was employed to investigate the decomposition temperature of MgH_2_ powder samples and their corresponding samples doped a-Zr_2_Cu powders that were obtained after different RBM time. Besides, this technique was used to identify the crystallization behavior of a-Zr_2_Cu powders. The DSC procedure and analysis were shown elsewhere^45–47^.

#### Hydrogenation/dehydrogenation kinetics behavior

The absorption/desorption kinetics of MgH_2_ powders obtained after different RBM times were investigated at 250 °C through Sievert’s method using PCT-Pro2000 (Setaram–France). The applied hydrogenation and dehydrogenation pressures were 10 bar and 400 mbar, respectively (see Refs.^23,24,42,43^).

## Supplementary information

Supplementary Information

## Data Availability

All datasets reported in this manuscript are available from the corresponding author on a reasonable request.
