# Supplementary material for: From gangue to the fuel-cells application
Source: Sci Rep. 2020 Nov 18;10:20022. doi: 10.1038/s41598-020-76503-6 (PMC7676253; doi:10.1038/s41598-020-76503-6)
Supplement: Supplementary file 1 — Supplementary Information [file 41598_2020_76503_MOESM1_ESM.pdf]

Supplementary Files

From gangue to the fuel-cells application

M. Sherif El-Eskandarany[1,2,*], Sultan Majed Al-Salem[1,3], Naser Ali[1,4], Mohammad Banyan [1,2,+], Fahad Al-Ajmi[1,2,+], and Ahmed Al-Duweesh[1,2,+]

[1]Kuwait Institute for Scientific Research, 13109, Kuwait

[2]Energy and Building Research Center

[3]Ocid: 0000-0003-0652-4502

[4]Ocid: 0000-0001-5931-0335

[+]These authors contributed equally to this work

*Corresponding; msherif@kisr.edu.kw ; orcid: 0000-0003-1851-7553

Keywords: Solid-waste metals; Hydrogen storage; plastic deformation; reactive ball milling.

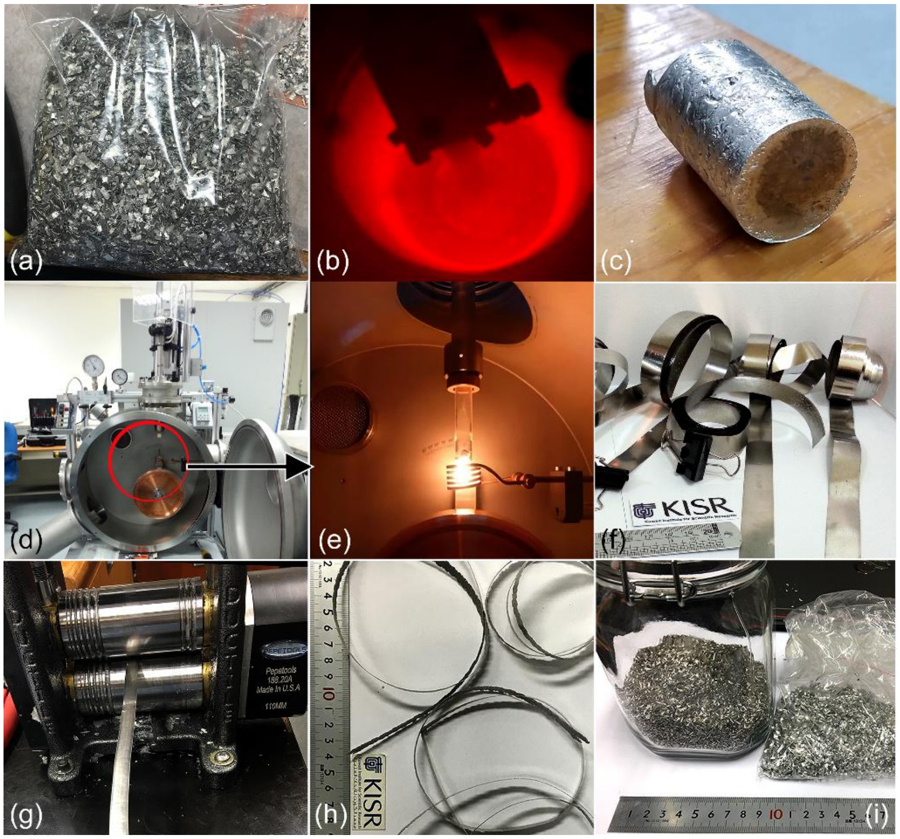

Fig. S1. Conversion of solid-waste (SW) Mg machining chips into macroscale Mg-shots. (a) as-received SW-Mg chips, (b) melting and casting processes, (c) as-casted pure (99.95 wt%) Mg-ingot, (d), (e) melt spinning (MS) process, (f) as -MS Mg-ribbons, (g) cold rolling (CR) process, (h) as-CR Mg-strips, and (i) as-snipped Mg -shots.

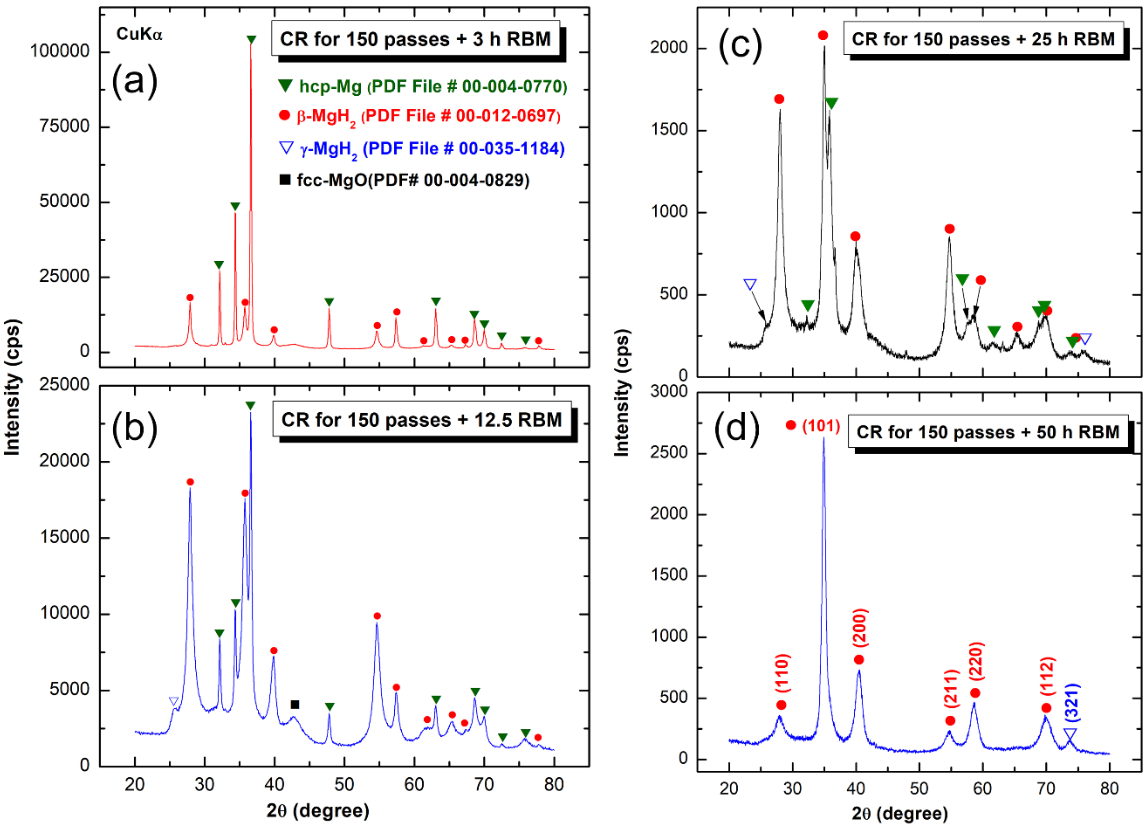

Fig. S2. Effect of RBM time on the crystal structure of Mg ribbons CR for 150 passes. The CR ribbons were snipped into small pieces and then RBM under 15 bar of H_2 for (a) 3 h, (b) 12.5 h, (c) 25 h, and (d) 50 h.

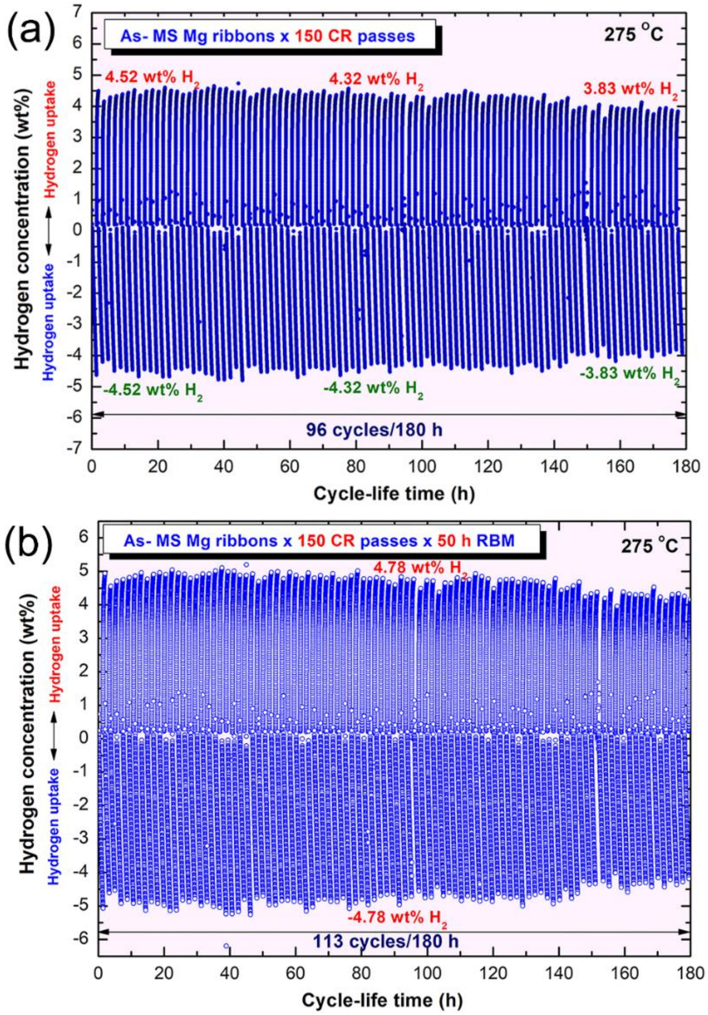

Fig. S3. Cycle-life-time of (a) as –CR Mg ribbons for 150 passes, and (b) as –CR for 150 passes followed by 50 h of RBM under 50 bar of H2 gas pressure. The measurements were conducted at 275oC under hydrogenation and dehydrogenation pressure of 10 bar and 400 mbar, respectively.

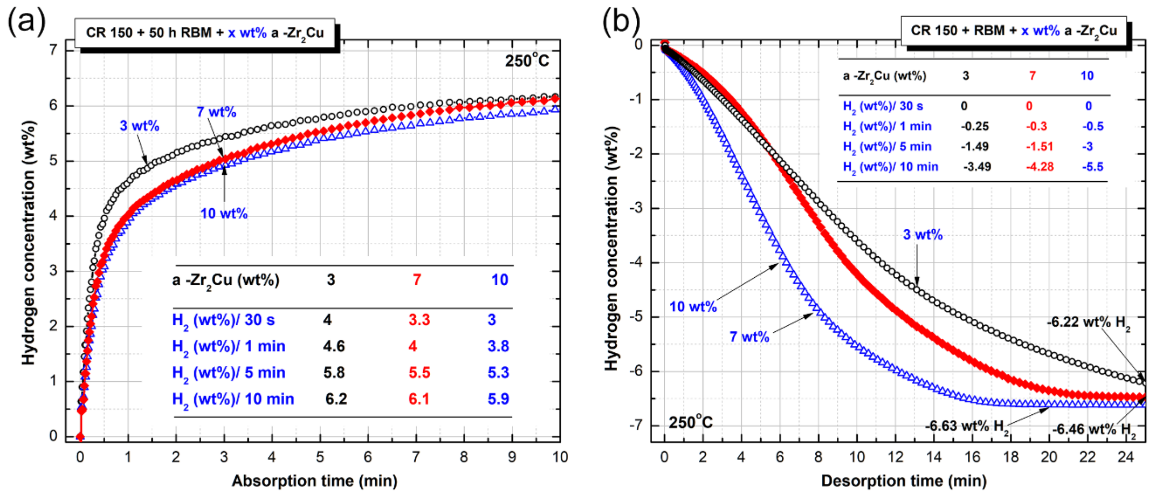

Figure S4. Effect of a –Zr_2Cu concentration (x, wt%) on the (a) hydrogenation, and (b) dehydrogenation kinetics behavior of MgH_2/ x wt% a –Zr_2Cu (x; 3, 7, and 10 wt%) powders obtained after 50 h of RBM time. The measurements were conducted at 250°C under uptake and release H_2 pressure of 10 bar and 400 mbar, respectively.
